# Learning meaningful representation of single-neuron morphology via large-scale pre-training

**DOI:** 10.1093/bioinformatics/btae395

**Published:** 2024-09-04

**Authors:** Yimin Fan, Yaxuan Li, Yunhua Zhong, Liang Hong, Lei Li, Yu Li

**Affiliations:** Department of Computer Science and Engineering, The Chinese University of Hong Kong, Hong Kong, 999077, China; Harbin Institute of Technology, Shenzhen, Guangdong, China; Nanjing University, Nanjing, Jiangsu, China; Department of Computer Science and Engineering, The Chinese University of Hong Kong, Hong Kong, 999077, China; Department of Computer Science and Engineering, The Chinese University of Hong Kong, Hong Kong, 999077, China; Department of Computer Science and Engineering, The Chinese University of Hong Kong, Hong Kong, 999077, China

## Abstract

**Summary:**

Single-neuron morphology, the study of the structure, form, and shape of a group of specialized cells in the nervous system, is of vital importance to define the type of neurons, assess changes in neuronal development and aging and determine the effects of brain disorders and treatments. Despite the recent surge in the amount of available neuron morphology reconstructions due to advancements in microscopy imaging, existing computational and deep learning methods for modeling neuron morphology have been limited in both scale and accuracy. In this paper, we propose MorphRep, a model for learning meaningful representation of neuron morphology pre-trained with over 250 000 existing neuron morphology data. By encoding the neuron morphology into graph-structured data, using graph transformers for feature encoding and enforcing the consistency between multiple augmented views of neuron morphology, MorphRep achieves the state of the art performance on widely used benchmarking datasets. Meanwhile, MorphRep can accurately characterize the neuron morphology space across neuron morphometrics, fine-grained cell types, brain regions and ages. Furthermore, MorphRep can be applied to distinguish neurons under a wide range of conditions, including genetic perturbation, drug injection, environment change and disease. In summary, MorphRep provides an effective strategy to embed and represent neuron morphology and can be a valuable tool in integrating cell morphology into single-cell multiomics analysis.

**Availability and implementation:**

The codebase has been deposited in https://github.com/YaxuanLi-cn/MorphRep.

## 1 Introduction

Cell morphology is a crucial phenotype in single-cell biology as it is closely related to gene expression, cell types, and spatial context. Neurons, which are specialized cells in the nervous system, contain complex structures including cell body, dendrites and axon, while neurons in different parts of the nervous system and different species have diverse morphology structure. Understanding and modeling single-neuron morphology is key to determining neuron type and function, linking different omics and data modalities in the nervous system and identifying disease mechanism. With the recent advancement in imaging technologies, it is now possible to obtain high-resolution microscopic images for neurons and glia in the nervous system. Based on the morphological reconstructions, researchers have developed a wide range of neuron tracing methods to digitally reconstruct the 3D structure of neurons from microscopic images. The reconstructed 3D structure of neurons contain the 3D position and radius of elements in the neurons, including soma, axon and dendrite, as well as their connectivity information.

Learning robust and meaningful representation of neuron morphology reconstructions remains a formidable challenge till now. The first issue arises from the high noisy level in neuron reconstructions. The process of acquiring microscopic images may introduce noise and artifacts that can affect the quality and fidelity of the data. Though the neuron 3D structure reconstruction with neuron tracing methods has removed some noise and artifacts in the raw images, the developed computation methods still need to be robust enough to noises. Secondly, neuron reconstructions have complex 3D structures, thus applying deep learning methods to model neuron reconstructions are more challenging compared with sequence data such DNA and protein. Lastly, compared with single-cell sequencing, neuron morphology reconstruction requires much more time and money and for now it is still technically challenging and costly to simultaneously reconstruct neuron morphology at scale. Thus, much less data for neuron morphology is available. Developing deep learning methods for neuron morphologies is therefore challenging as these methods usually learn patterns from large scale data.

Several computational methods, including both statistical and deep learning methods, have been developed to obtain neuron morphology representation. Schubert *et al.* (2019) proposes to train cellular morphology learning networks (CMNs), a multi-view variant of CNN to infer low-dimension neuron embeddings. Though this method can generate meaningful neuron embeddings for cell-type identification, it requires multiple view projection of 3D neuron morphologies, which leads to information loss in the projection process. MorphVAE (Laturnus and Berens 2021) proposes to generate low dimension neuron embedding through training a variational autoencoder on the random walks of the morphology tree. The major limitations of MorphVAE include the low computational efficiency and sub-optimal representation learning performance. CAJAL (Govek et al. 2023) proposes to use Gromov–Wasserstein (GW) distance between neurons to embed neuron morphology. CAJAL has high interpretability compared with other methods and the output embeddings are highly discriminative of neuron morphology shapes. However, the scalability of CAJAL is poor. As distance matrix computation across neurons is required to obtain neuron embeddings, it is not feasible to apply CAJAL to large scale neuron morphology datasets. Recently, there are a series of new methods focusing on neuron morphology representation learning, including MorphGNN (Zhu *et al.* 2022), TreeMoco (Chen *et al.* 2022), MACGNN (Zhao *et al.* 2022), and GraphDino (Weis *et al.* 2022, 2023). These methods are based on graph neural networks and are trained with contrastive learning loss to learn a meaningful low dimension embedding. While these methods can usually perform accurate neuron type classification and clustering, the scale of datasets used by these methods is rather small, usually with no >2000 neurons, which limit the scalability and accuracy of these methods. DSM (Xiong *et al.* 2024) is a deep sequential model for neuron morphology which also only utilizes thousands of datasets. Several other works, including MorphOcc (Hansel *et al.* 2023) and MorphGrower (Yang *et al.* 2024), have been proposed to generate neuron morphology with generative models, but the performance of neuron morphology embedding may be suboptimal.

To this end, we propose MorphRep, which learns meaningful representation of neuron morphology via large-scale pre-training. We collected over 250 000 neuron reconstructions for >90 species from existing database, which are several orders of magnitude higher than the dataset size of existing methods. To mitigate the negative impact of reconstruction variability and noise in the dataset, we used extensive data augmentation strategies to enhance the robustness of MorphRep. By leveraging graph transformer networks and self-supervised representation learning, MorphRep is capable of generating high-quality neuron morphology embeddings at scale. To address the challenge of data scarcity in downstream applications, we proposed a novel transfer learning strategy for fine-tuning MorphRep. This involves incorporating lightweight adapter modules in each transformer layer, freezing the pre-trained parameters, and fine-tuning only the adapter and classifier parameters. To demonstrate the effectiveness of MorphRep, we evaluated MorphRep on a wide range of downstream applications. Firstly, we assessed MorphRep using several established benchmarking datasets commonly used in existing methods, including neuron cell type classification and neuron brain region classification. Our results consistently showed that MorphRep achieves state-of-the-art performance in both unsupervised and supervised classification settings, outperforming baseline methods. Then, we demonstrated the capabilities of MorphRep to characterize neuron shape at atlas-level. We observed that neurons with distinct morphometric features, such as height or number of branches, are distributed into distinct clusters within the MorphRep embedding space. Furthermore, we revealed the relationship between neuron properties including cell types and brain regions with MorphRep embeddings. Given the hierarchical annotations of neuron types (primary cell type, secondary cell type, and tertiary cell type), we found that MorphRep can generate fine-grained embeddings that provide clear separation of tertiary cell types. Moreover, MorphRep embeddings also exhibit variations among neurons of different ages and brain regions, demonstrating its ability to encode comprehensive information regarding neurons from multiple perspectives. Apart from its impressive performance on benchmarking datasets and its capability to characterize neuron properties at an atlas-level, one prominent application of MorphRep is the ability to distinguish neurons under various conditions, including diseases, environmental factors, drug injections, and genetic perturbations. While a substantial amount of neuron morphology condition datasets have been released through wet lab efforts, few studies have explored the automatic classification of neuron conditions based on morphology. We evaluated the classification performance of MorphRep on 20 neuron morphology condition datasets and validated the superiority of MorphRep over baseline methods.

## 2 Materials and methods

### 2.1 Pre-training data curation

We collected neuron morphology reconstructions from a centrally curated inventory of digitally reconstructed neurons and glia called NeuroMorpho.Org (https://neuromorpho.org/). We collected over 250 000 neuron reconstructions along with their metadata including but not limited to species, brain region, cell types, and reconstruction softwares. All neuron reconstructions are in swc file format, which is a standardized format in storing digital neuron reconstructions. Based on the neuron reconstruction, we represented each neuron as a tree, with each node containing features include the spatial coordinates, node type and node radius. Each node is connected with its parent node and children node in the neuron. Details on the swc file format and the advantages of using graph-structured swc file format for the analysis of neuron morphology over original 3D images in our and previous studies can be found in Supplementary Appendix S1.1. We filtered out neurons that exist in the benchmarking dataset in our downstream study.

### 2.2 Data preprocessing and augmentation

We first centered all nodes in the neuron by subtracting the coordinates of the first node (usually soma). Neuron node coordinates, radius, and type are then concatenated as the node feature. As some neuron reconstruction softwares may over-sample on the axon of neurons, it is possible that for some neuron reconstructions, >80% of the nodes in the neuron are axons. Existing works (Chen *et al.* 2022, Weis *et al.* 2023) directly removed all axons considering their sample redundancy. We removed the axon nodes which only have one or two neighbor nodes and kept the branching axon nodes. We believed these nonbranching nodes contain limited effect on overall topology thus removing these nodes will cause little information loss and the computational efficiency can be greatly improved. As mentioned in Section 1, neuron reconstructions may be noisy, thus various data augmentation techniques should be used to enhance the robustness of our method. Data augmentation techniques used in this paper can be divided into two categories: (i**)** shape augmentation and (ii**)** topology augmentation. The shape augmentation contains shifting and rotation augmentation. Shifting means adding random noise to the 3D coordinates of neuron nodes. Rotation means randomly rotating the neurons along a random axis. The topology augmentation contains branch deletion and subgraph sampling. Branch deletion means randomly deleting branches in the neuron while subgraph sampling means randomly sampling nodes from the original graph. All data augmentation techniques did not affect the connectivity of the graph.

### 2.3 Backbone architecture

Our model backbone is based on graph transformers. The network input is the neuron morphology represented as an undirected graph G={V,A}, where $V={\left\{v_{i},x_{i}\right\}}_{i=1}^{n}$ contains nodes from the neuron. The node feature of *v_i_* is represented by $x_{i}$. $A={\left\{e_{ij}=(v_{i},v_{j})\right\}}_{i,j\in (1,n)}$. The [CLS] token $v_{\text{CLS}}$ is added to the input with randomly initialized node feature and position embedding and the [CLS] token representation in the last layer is used as the final graph representation. The [CLS] token is also added to the adjacency matrix with weight 1 to itself and 0 to other nodes in the graph. The graph transformers contain an embedding layer and a series of encoder layers. In the embedding layer, the original node feature embeddings are projected to a higher-dimension feature embedding through two MLP layers. Following Dwivedi *et al.* (2022), we use the normalized graph Laplacian matrix **L** as position encoding, which is computed by $L=I-D^{-1/2}AD^{-1/2}=U^{T}\Lambda U$, where **I** is the identity matrix, **D** is the *N *×* N* degree matrix, **A** is the adjacency matrix, **U** is the eigenvector matrix and Λ is the eigenvalue matrix. The position encoding and the node embeddings are added up and fed into subsequent encoder layers. Following the traditional Transformers (Devlin et al. 2019) setting, Each encoder layer contains layer normalization, graph attention, layer normalization, and MLP layers sequentially. Inspired by GraphDino (Weis *et al.* 2023), the attention matrix in graph attention is computed as follows: $\text{Attention}(Q,K,V,A)=\sigma (\lambda \frac{QK^{T}}{\sqrt{d_{k}}}+\gamma A)V$. The key **K**, query **Q** and values **V** are computed from the projection of the token embeddings. $[Q,K,V]=W_{1}x$. **A** is the adjacency matrix of the input neuron graph. *λ* and *γ* are also projected embeddings from token embedding $\left[\lambda ,\gamma ]=\text{exp}(W_{2}x\right)$. The designed graph attention mechanism can be considered as a mixture of vanilla transformer attention and message-passing algorithms controlled by the magnitude of *λ* and *γ*.

### 2.4 Pre-training strategies

Recent advances in self-supervised pre-training on images (Oquab *et al.* 2023) in computer vision have shown that it is possible to learn robust and meaningful low-dimension feature without explicit label supervision. We developed a pre-training strategy on neuron morphology data without label supervision. Specifically, the model contains teacher network and student network. Each network is based on graph transformers as described in Section 2.3. The model is trained by multiple loss terms, including consistency loss, reconstruction loss and KoLeo regularization loss, enabling high-quality representation learning at multiple levels including graph, sub-graph, and nodes. Due to the space limits, the details of the pre-training strategies are described in Supplementary Appendix S1.2 and the hyperparameters in pre-training are described in Supplementary Appendix S1.3.

### 2.5 Fine-tuning strategies

Many neuron morphology datasets contain a very limited number of neurons as they may focus on a specific region in the nervous system. In that case, directly fine-tuning MorphRep on these datasets may lead to over-fitting and sub-optimal performance. To resolve this issue, we used a transfer learning strategy to enable effective and efficient fine-tuning of MorphRep on small downstream datasets. Concretely, we added an adapter module within each graph transformer layer. The input is passed through the original layer and adapter simultaneously and the outputs from these two parts are added up as the final output. During the fine-tuning process, only the parameters in the adapters and the classifier are updated, which can reduce the possibility of over-fitting as only a small number of parameters are updated. More details with respect to fine-tuning strategies are described in Supplementary Appendix S1.4.

### 2.6 Experiment settings

**Figure 1.**
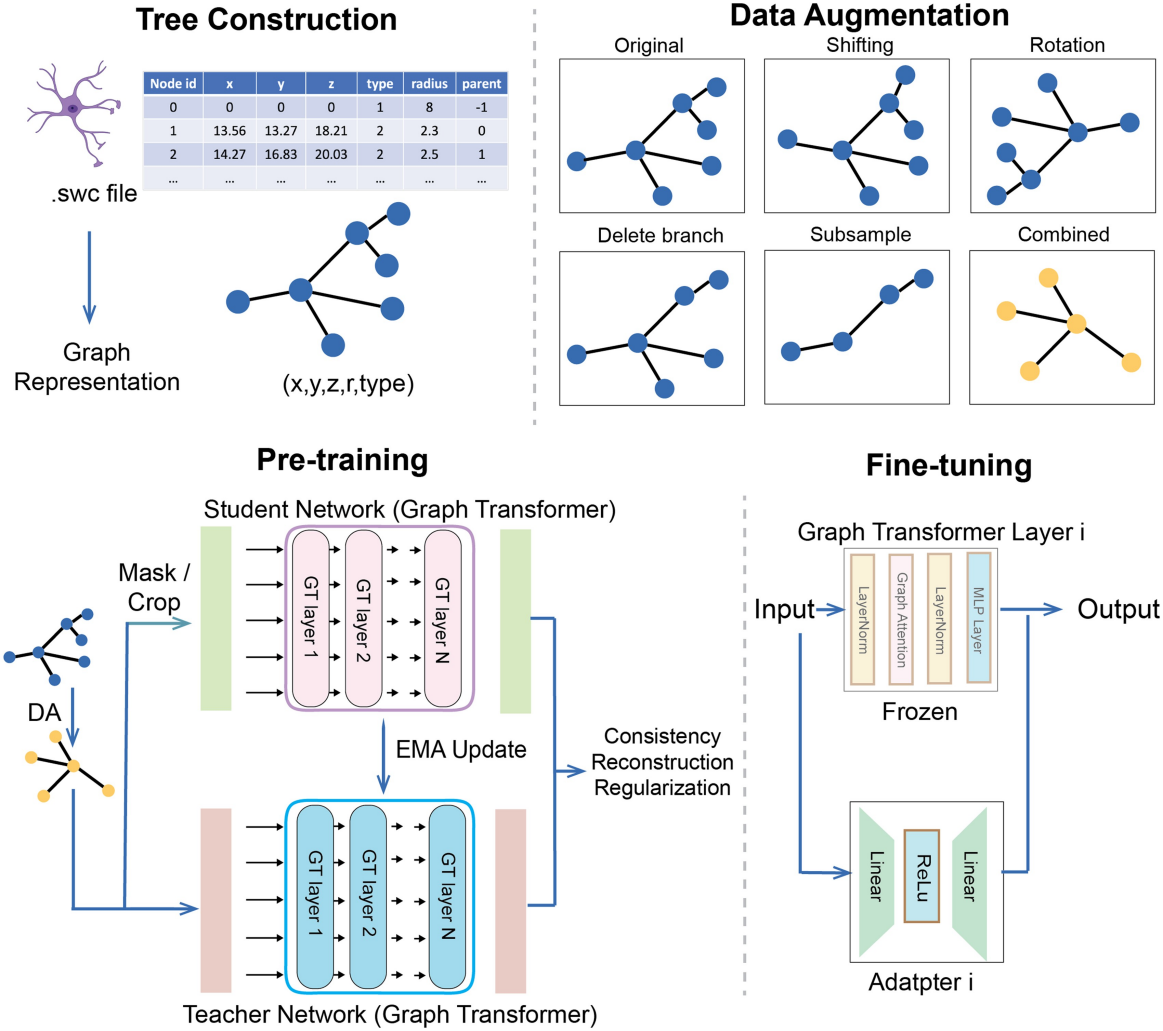
MorphRep data processing and training pipeline. **Top-Left**: We constructed tree-structured data from neuron reconstruction files. **Top-Right**: Data augmentation on the neuron morphology. **Bottom-Left**: Pre-training steps of MorphRep. The input data is first passed to the data augmentation module (DA). The augmented view is sent to the teacher network, which is based on graph transformer. The augmented view is masked or cropped and then sent to the student network with the same architecture. The student network output and the teacher network output are used to compute consistency loss, reconstruction loss and regularization loss to optimize the parameters. The teacher network is updated using exponential moving average of the student network. **Bottom-Right**: When fine-tuning MorphRep on downstream tasks including neuron cell type classification, we add an adapter module inside each transformer layer. The input is passed to the original transformer layer modules and adapter module at the same time. The pre-trained transformer parameters are frozen and only the adapter parameters are updated. Figure created with Biorender.com.

Following previous works in neuron morphology representation learning (Chen *et al.* 2022, Weis *et al.* 2023), we used seven commonly used datasets to benchmark the performance of MorphRep, namely ACT (Cell Type), ACT (Brain Region), BIL (Cell Type), BIL (Brain Region), M1-EXC (Cell Type), M1-EXC (Brain Region), and BBP. These datasets come from existing public available databases include M1-EXC (Scala *et al.* 2021), BBP (Ramaswamy *et al.* 2015), ACT (Gouwens *et al.* 2019), and BIL (Peng *et al.* 2021). The labels of these datasets are either cell types or brain regions. Some datasets are with the same neurons but have different label annotation. For instance, ACT (Cell Type) and ACT (Brain Region) contain the same neurons but are annotated differently based on cell types or brain regions. Details of these datasets are described in Supplementary Table S2. Apart from these commonly used benchmarking datasets, we also curated a number of neuron morphology condition classification datasets. Each neuron morphology condition classification dataset contain neurons from the control group and the condition group. The details of these datasets are in Supplementary Tables S3–S6. We compare MorphRep with several baseline methods in modeling neuron morphology, including CAJAL (Govek *et al.* 2023), MorphVAE (Laturnus and Berens 2021), and GraphDino (Weis *et al.* 2023). It is important to note that all these baseline methods are not pre-trained on large scale datasets and are only trained on thousands of neurons originally. To benchmark the performance of MorphRep on atlas-level neuron property characterization, we pre-trained GraphDino using the same dataset as MorphRep, and we denote this model as GraphDino-P and the original model as GraphDino-B. Due to the low scalability of CAJAL of MorphVAE, we did not train them on our pre-training datasets (Fig. 1).

## 3 Results

### 3.1 MorphRep achieves state-of-the-art performance on benchmarking datasets

**Figure 2.**
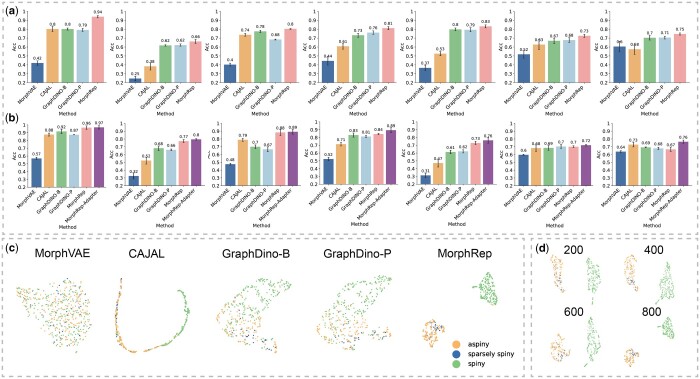
The performance of MorphRep on a wide range of benchmarking datasets. (a) Unsupervised classification performance of MorphRep and baseline methods on benchmarking datasets [from left to right: ACT (Cell Type), ACT (Brain Region), BIL (Cell Type), BIL (Brain Region), BBP, M1-EXC (Cell Type), M1-EXC (Brain Region)]. (b) Supervised classification performance of MorphRep and baseline methods on benchmarking datasets. (c) Visualization of the pre-trained MorphRep embeddings and baseline method embeddings for ACT (Cell Type) dataset. (d) MorphRep embeddings with different neuron sub-sampling strategies. neurons are downsampled ranging from 200 to 800 nodes on ACT (Cell Type) dataset. Metrics are computed using five random runs with random train/test split and we report the mean value and the standard deviation.

To evaluate the performance of MorphRep, we collected seven widely used and publicly available neuron morphology datasets to benchmark its performance against baseline methods. We assessed MorphRep in both unsupervised and supervised settings. In the unsupervised setting, we froze the parameters of MorphRep and extracted neuron morphology embeddings. We utilized a k nearest neighbor classifier to assign label annotations to classify neuron morphology. In the supervised setting, we fine-tuned MorphRep on specific datasets. For the baseline methods, we trained CAJAL and MorphVAE on the target datasets to obtain neuron embeddings in both settings, as they are not transferable due to their design. In Fig. 2a, we benchmarked the unsupervised classification performance of MorphRep and baseline methods on seven datasets and find that MorphRep consistently outperforms existing baselines. Among all the methods, MorphVAE performs worse, possibly due to its design of modeling the random walks in the neuron morphology graph instead of the entire morphology. In Fig. 2b, we benchmarked the supervised fine-tuning performance of MorphRep on benchmarking datasets. We observed that MorphRep achieves state-of-the-art performance among all methods. In addition, MorphRep-Adapter, which utilizes a lightweight adapter during fine-tuning (Section. 2.5), demonstrates competitive performance compared with MorphRep, with the additional benefit of lower fine-tuning costs. On small-scale datasets like M1-EXC, as conventional fine-tuning is likely to over-fit, MorphRep-adapter outperforms MorphRep by a larger margin. In Fig. 2c, we visualized the neuron morphology embeddings of ACT (cell type) dataset by projecting the neurons onto the UMAP space. It is evident that MorphVAE does not capture meaningful embeddings, as neurons from different cell types and brain regions are mixed together in the embedding space. In contrast, CAJAL embeddings exhibit distinguishable clusters corresponding to different cell types and brain regions. However, the ring-like distribution of CAJAL embeddings may pose challenges for clustering analysis. On the other hand, both GraphDino-B and GraphDino-P effectively separate neurons with distinct properties into distinct regions within the embedding space. Notably, MorphRep demonstrates exceptional performance by clearly distinguishing neurons into separate clusters, with neurons of the same cell type forming dense clusters. This highlights the strong expressive power of MorphRep. Additional visualizations of other neuron morphology datasets are provided in the Supplementary Fig. S4 with consistent conclusion. In Fig. 2d, we assessed the robustness of MorphRep when applied to neurons with different sub-sampling strategies. Neurons reconstructed using different imaging technologies and software may exhibit variations in the number of nodes. In addition, reducing the number of nodes within neurons can reduce the computational costs. Consequently, we downsampled the number of nodes in the neurons from the ACT (Cell Type) dataset ranging from 200 to 800 nodes. We observe that as the number of nodes in the neurons increases, the clusters formed by different cell types become denser and more distinct. However, even with as few as 200 nodes, MorphRep is capable of generating high-quality neuron embeddings that effectively differentiate cell types. In certain application scenarios where computational efficiency is crucial (such as real-time retrieval of similar neurons), MorphRep can generate robust and high-quality embeddings by downsampling the input nodes without sacrificing model performance.

### 3.2 MorphRep accurately characterize neuron shapes

**Figure 3.**
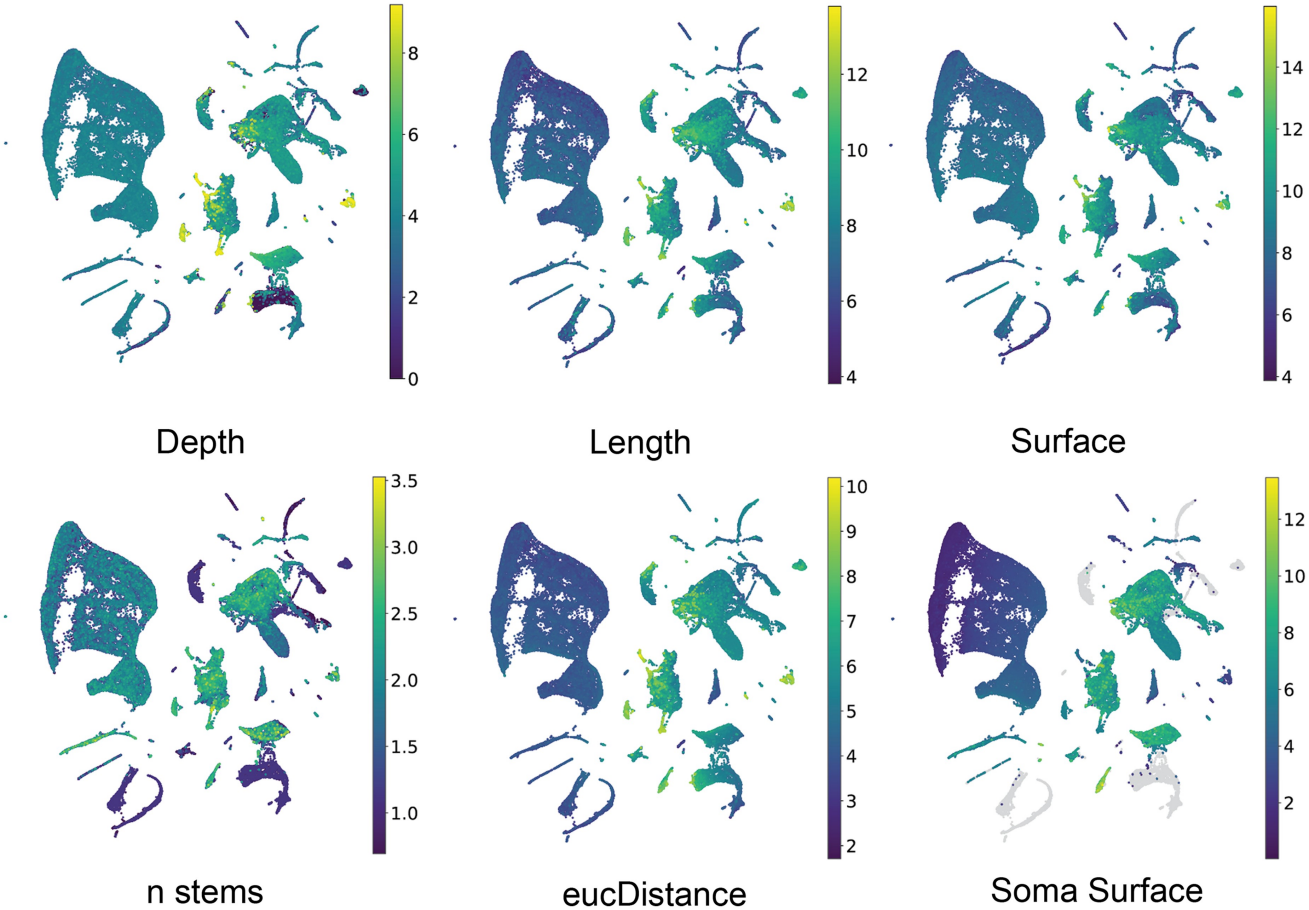
The characterization of MorphRep on neuron shape space. UMAP visualization of pre-trained MorphRep embeddings with respect to a wide range of neuron morphometric features. Neurons with distinct morphometric features form distinct groups in the MorphRep embedding space.

We then investigated whether the learned embeddings from MorphRep can effectively capture neuron shape properties. Given that MorphRep learns embeddings directly from the graph representation of neuron shapes, it is expected that these embeddings should be closely related to neuron shapes. Traditionally, researchers have used hand-crafted features known as morphometrics to quantify various shape properties of neurons (Wu *et al.* 2004). These morphometrics encompass a range of characteristics, including but not limited to neuron depth, branching degree, and surface area. In Fig. 3b, we presented the visualization of the UMAP embedding of pre-trained MorphRep embeddings with different morphometrics. The raw morphometric values are log-transformed to enable better visualization. We observed that neurons exhibiting distinct morphometrics tend to occupy separate regions within the UMAP embedding space. For instance, neurons with greater depth primarily cluster in the bottom-right region of the UMAP space, while neurons with longer lengths are concentrated in the right region compared to those in the left region. Furthermore, neurons with differing Euclidean distances, soma surface, and number of stems from distinct clusters within the embedding space. For comparison, we also visualized the UMAP embeddings of MorphRep and GraphDino-P embeddings on a wider range of morphometrics in Supplementary Fig. S1. The embeddings from GraphDino-P demonstrated notably less discriminative properties concerning morphometrics compared with MorphRep embeddings. Specifically, it is challenging to identify clusters of neurons with high diameter and high branch order from GraphDino-P embedding. Detailed information on the meaning of these morphometrics is shown in Supplementary Appendix S1.5.

### 3.3 MorphRep generates meaningful embeddings related to cell type and brain region

**Figure 4.**
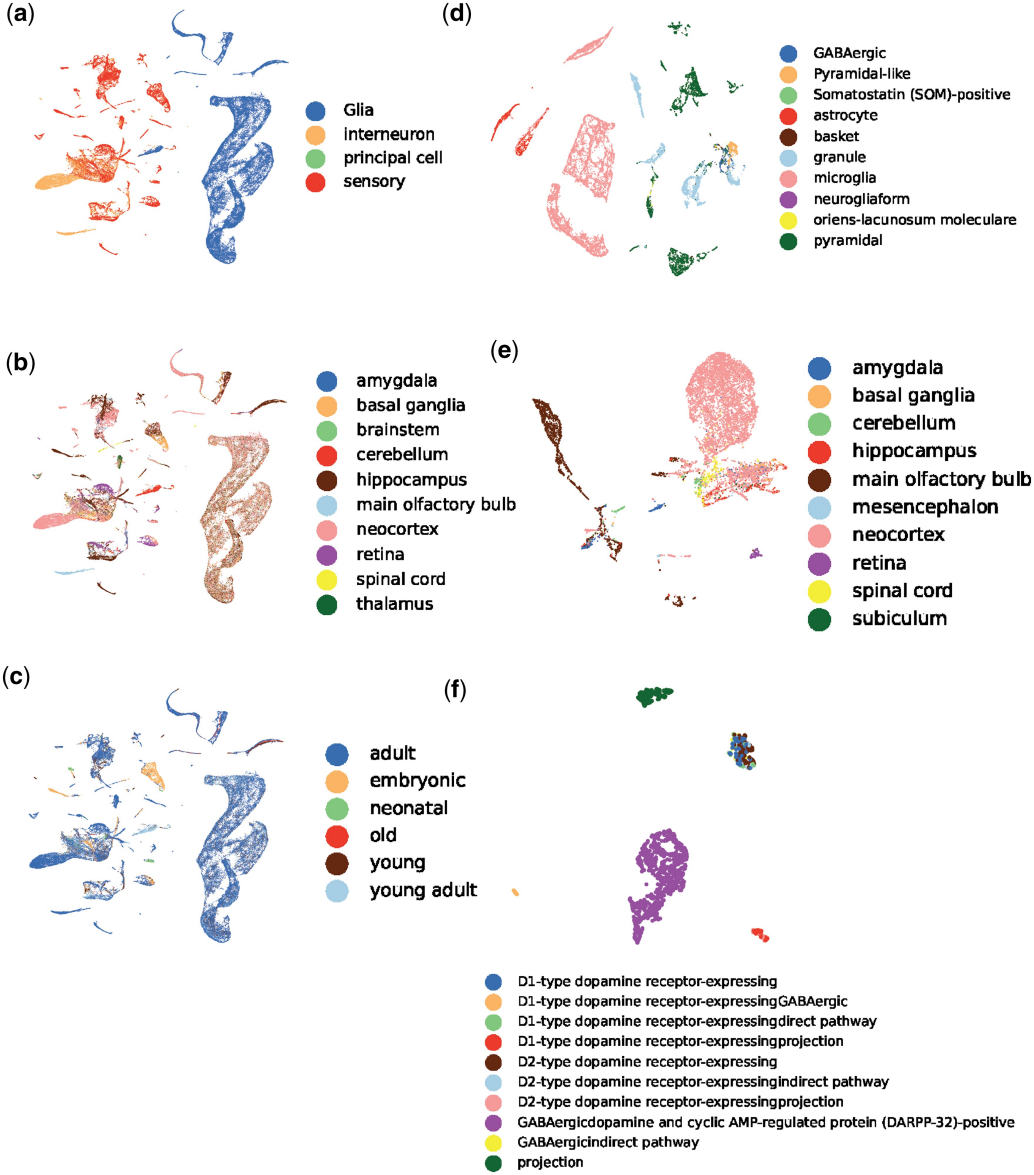
UMAP visualization of neuron morphology embeddings generated by MorphRep. A(B) denotes the visualization of neurons in A colored by B. (a) Mouse (primary cell type). (b) Mouse (primary region). (c) Mouse (age). (d) Mouse hippocampus (secondary cell type). (e) Mouse interneuron (primary brain region). (f) Mouse principal cell-medium spiny cell (tertiary cell type). An extended version of this figure is Supplementary Fig. S2.

We then presented compelling evidence that MorphRep, even in the absence of supervision, can learn neuron morphology embeddings that effectively capture meaningful properties associated with neurons at atlas-level. These properties include but are not limited to cell types, brain regions and ages. It is worth noting that neuron type annotations exhibit a hierarchical structure, wherein a neuron possesses primary, secondary, and tertiary cell types. We first visually depicted the neuron properties (primary cell types, primary brain regions, and ages) of mouse neurons in Fig. 4a–c. We observed that each cluster in the embedding space corresponds to a specific primary neuron type. Notably, multiple clusters may exist for a single primary neuron type, as neurons within that category may differ in terms of secondary cell type and brain regions. Similarly, as shown in Fig. 4b and c, respectively, neurons belong to different brain regions and ages also form distinct clusters in the embedding space. We also provided visualization on other species including human, drosophila and rat in Supplementary Fig. S2 to demonstrate the consistency of our findings across species. Subsequently, we turned to a more detailed analysis by examining neurons within a specific region or specific secondary and tertiary cell types within a given species. In Fig. 4d, we chose mouse hippocampus in mouse and visualized the neuron morphology embeddings based on the secondary cell types. It can be clearly observed that within one specific brain region, neurons belong to different secondary cell type clearly separate with each other. As neurons belong to one cell type in different brain regions may have different functions or connectivity patterns, their morphologies may also have substantial differences. Therefore, in Fig. 4e, we observed that neurons of the same cell type exhibit varied distributions depending on their brain regions. Even within a specific secondary cell type, multiple distinct clusters still exist, as depicted in Fig. 4d. To conduct a more refined analysis, we explored the distribution of tertiary cell types within a particular secondary cell type. In Fig. 4f, we revealed that MorphRep effectively discriminates neurons with different tertiary cell types. For instance, in the case of mouse principal cell-medium spiny cell, neurons with dissimilar pathways and receptors form distinct groups. This finding emphasizes the remarkable expressive power of MorphRep in identifying subtle variations in neuron morphology, considering that neurons of the same secondary cell type may already exhibit substantial similarity. We provided more visualization across different cell types and brain regions in Supplementary Fig. S2 with respect to Fig. 4d–f to fully demonstrate effectiveness of MorphRep and the consistency of our conclusion.

As comparison, we also visualized the embeddings of GraphDino-P with respect to a wide range of neuron properties in Supplementary Fig. S3. It can be observed from Supplementary Fig. S3a–i that embeddings from GraphDino-P can distinguish between neurons with different primary cell types, but fail to distinguish between different brain regions and ages. Moreover, though GraphDino-P can separate neurons belonging to different secondary cell types within one specific brain region (Supplementary Fig. S3j–m), it fails to separate neurons from one primary cell type based on different brain regions (Supplementary Fig. S3n–q). In conclusion, MorphRep proficiently captures fine-grained distinctions relating to neuron hierarchical cell types, brain regions, and ages, thereby enabling comprehensive characterization of neuron properties.

### 3.4 MorphRep efficiently diagnosis neurons under various conditions

**Figure 5.**
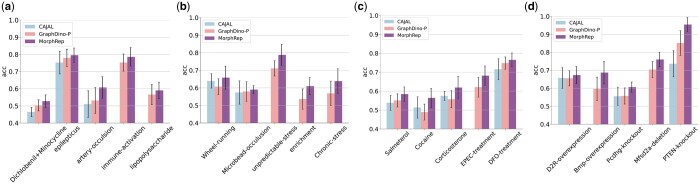
Classification performance of pre-trained MorphRep embeddings on several neuron morphology condition datasets. (a) Disease. (b) Environment. (c) Drug injection. (d) Genetic perturbation. We run each experiment with five different random seeds and train/test split. Some results for CAJAL are missing due to the scalability of this method on large scale datasets. Metrics are computed using five random runs with random train/test split and we report the mean value and the standard deviation.

Different cellular responses emerge when cells are exposed to various perturbations, such as chemical compounds, genetic edits, and changes in the environment. While several existing studies (Bardy *et al.* 2016, Roohani *et al.* 2023) have used deep learning methods to model shifts in gene expression profiles and cell morphology under different conditions, including drug and genetic perturbations, there are limited investigations on the response of neurons to diverse perturbations. Due to the intricate 3D structures of neurons, their responses to perturbations can be intriguing and not easily distinguishable. Therefore, we evaluated the zero-shot classification capabilities of MorphRep on a wide range of neuron morphology datasets with various condition factors, including drug injection, genetic editing, environment change, and disease. Each neuron morphology dataset with condition factors comprises control neurons as well as neurons with specific perturbations. We trained a k-nearest-neighborhood classifier on each neuron morphology dataset to predict the neuron’s condition (control or with perturbation) based on pre-trained embeddings from MorphRep. The train-test split ratio is set to 7:3 for each dataset. To tackle the data imbalance issue between class labels in the training set, we utilized SMOTE (Chawla *et al.* 2002) to oversample the minority class and undersample the majority class in the training set. In Fig. 5, we demonstrated the performance of embeddings from MorphRep in distinguishing neuron conditions across 20 neuron morphology datasets encompassing disease conditions, environment conditions, drug injection and genetic perturbations. These neuron condition datasets are collected from neuronmorph.org. For baseline comparison, we trained CAJAL model on each dataset separately and use pre-trained GraphDino-P to get the neuron embeddings. We found that the pre-trained embeddings from MorphRep exhibit the best classification performance across multiple neuron conditions, with classifier accuracy consistently outperforming baseline methods including CAJAL and GraphDino-P. The classifier metrics on the validation set of most dataset are >0.6, which is unexpected since neurons under different conditions only display subtle structural changes and are typically distinguishable solely by domain experts. Several datasets contain over 10 labels (e.g. Bmp-overexpression). We provided detailed information related to these datasets in Supplementary Tables S3–S6.

## 4 Discussion

The emergence of large scale neuron morphology datasets across species poses challenges to traditional statistical approaches and existing deep learning based approaches for neuron morphology analysis and calls for powerful and meaningful representation learning methods to mine the latent neuronal knowledge from the vast amount of neuron morphology datasets. To address these challenges, we proposed MorphRep, which leverages >250 thousand neuron morphology reconstructions for learning meaningful representation. We demonstrated the state-of-the-art performance of MorphRep on a wide range of neuron morphology datasets compared with existing methods. We also performed in-depth analysis on the neuron morphology space characterized by MorphRep. The application of MorphRep on identifying neuron conditions across disease, environment conditions, genetic perturbation and drug injections is also explored.

In the future, there are several potential directions to extend MorphRep. With the rapid development of patch-seq technologies, it is now possible to profile the morphology, gene expressions and electron-physics features of the same neurons simultaneously. We plan to extend MorphRep to integrate neuron morphology into single-cell multiomics analysis in the future. Meanwhile, current neuron morphology search are based on keywords, while MorphRep can be a scalable and efficient approach for neuron morphology dense retrieval. We anticipate that MorphRep has the potential to become a significant breakthrough in neuron morphology representation learning. Our study demonstrates its strong expressive capabilities, and we are committed to applying MorphRep to a wider range of tasks related to neuron morphology.

## Supplementary Material

btae395_Supplementary_Data

## Data Availability

The pre-training datasets were derived from sources in the public domain: https://neuromorpho.org/. The benchmarking datasets are available in https://github.com/YaxuanLi-cn/MorphRep/tree/main/benchmark_dataset.
