# Correction to: Learning meaningful representation of single-neuron morphology via large-scale pre-training

**DOI:** 10.1093/bioinformatics/btaf584

**Published:** 2025-11-13

This is a correction to: Yimin Fan, Yaxuan Li, Yunhua Zhong, Liang Hong, Lei Li, Yu Li, Learning meaningful representation of single-neuron morphology via large-scale pre-training, *Bioinformatics*, Volume 40, Issue Supplement_2, September 2024, Pages ii128–ii136, https://doi.org/10.1093/bioinformatics/btae395

The following changes have been made to the originally-published manuscript. Omitted supplementary material has been added to the paper.

## Supplementary Material

btaf584_Supplementary_Data

