# Supplementary material for: Learning meaningful representation of single-neuron morphology via large-scale pre-training
Source: Bioinformatics. 2024 Sep 4;40(Suppl 2):ii128–36. doi: 10.1093/bioinformatics/btae395 (PMC11373321; doi:10.1093/bioinformatics/btae395)
Supplement: btae395_Supplementary_Data [file point-by-point-response.pdf › point-by-point-response.pdf]

Review 1

Review comments

The article presents a method for learning morphological representations for neurons, coined MorphRep. The method is based on learning the representations from a large training set of 250000 neurons, and uses graph transformers for encoding the feature representation from graph-structured neuron morphology. The strength of the article is the extensive experimental part, where the proposed method is compared agains several alternative approaches, and the proposed method achieves state-of-the-art results. Implementation of the method is available.

As minor comments for improving the article: -To add confidence on the results, the authors could show or discuss the effect of umap parameters on the visualization. -

Response: Thanks for your valuable feedback to our work. We vary the parameters of UMAP for Fig.4 and analyze the impact of UMAP parameters on visualization. We varied the n_neighbors and min_dist parameters of the UMAP algorithm and visualized the neuron morphology embeddings. The min_dist changes from 0.1,0.2,0.3,0.4 to 0.5 and the n_neighbors changes from 5,10,20,30,40 to 50. The results are shown respectively in Supplementary Fig.5-9. We can see that the results and observations are generally consistent across different hyper-parameter settings, which demonstrates the generalizability and stability of our method.

Giving more thorough context for the study through citing relevant papers in the area is recommended.

Response: Thanks for your valuable feedback to our work. Due to the page limit, in our submission, we mainly discussed the papers used for baseline comparison in this area. We have added more thorough context and cited more relevant papers for the in this area in the introduction section of the paper. We also listed some of the papers here:

[1] Large-scale unsupervised discovery of excitatory 2 morphological cell types in mouse visual cortex. https://www.biorxiv.org/content/10.1101/2022.12.22.521541v1
[2] MorphOcc: An Implicit Generative Model of Neuronal Morphologies https://openreview.net/forum?id=D9SA02esgh
[3] A Synchronized Layer-by-layer Growing Approach for Plausible Neuronal Morphology Generation https://arxiv.org/pdf/2401.09500.pdf
[4] DSM: Deep sequential model for complete neuronal morphology representation and feature extraction. https://www.cell.com/patterns/pdf/S2666-3899(23)00298-2.pdf
[5] Graph Representation Learning for Large-Scale Neuronal Morphological Analysis https://ieeexplore.ieee.org/stamp/stamp.jsp?arnumber=9895206

Review 2

Review comments

In Fig. 3, the numbers on the color bars are not readable. The same for legends of Figure 4.

Response:

Thanks for your valuable feedback to our work. We have modified Fig. 3 and Fig. 4 accordingly.
