# Supplementary material for: Correction to: Learning meaningful representation of single-neuron morphology via large-scale pre-training
Source: Bioinformatics. 2025 Nov 13;41(11):btaf584. doi: 10.1093/bioinformatics/btaf584 (PMC12614739; doi:10.1093/bioinformatics/btaf584)
Supplement: btaf584_Supplementary_Data [file btaf584_supplementary_data.pdf]

1. Appendix

1.1. Details on the swc file format

swc file format is a standard format for storing neuron morphologies. The swc file contains 7 columns. Each row in the swc file indicates one node in the neuron. The detailed information of the 7 columns is: **Column 1**: Sample id, the id of the node in the neuron. **Column 2**: type id, the id of the node type. For example, 1 indicates soma and 2 indicates axon. **Column 3,4,5**: spatial coordinates (x,y,z) of nodes. **Column 6**: radius of node. **Column 7**: parent of node.

Compared with the raw 3D image of neuron, using swc file format to store neuron morphology has the following advantages.

- Data Size and Efficiency. The SWC format is a text-based representation of the neuron's morphology, which consists of a series of connected nodes and branches. It describes the location, radius, and connectivity of each neuronal compartment efficiently. In comparison, images, especially high-resolution 3D images, can take up much more space and are less efficient to process and analyze.
- Quantitative Analysis. SWC files allow for direct, quantitative analysis of the neuron's structure. Parameters like branch lengths, branch angles, dendritic and axonal tree complexity, and the distribution of synaptic sites can be measured and compared across different neurons. Images would require additional processing to extract such quantitative data.
- Scalability and Standardization. The SWC format provides a standardized way to represent neuronal morphology that can be easily shared, compared, and merged across datasets. This standardization is crucial for collaborative research and meta-analysis across different labs. Images would require a manual or semi-automated process to interpret and convert them into a comparable format. Different imaging platforms and reconstruction softwares in different labs will also lead to large gap in the original 3D images, but the standardized swc format can provide a uniform solution for data sharing and comparison.
- Interoperability with Simulation Software: Many computational neuroscience tools can directly import SWC files to create realistic neuron models for simulations. This interoperability is important for integrating experimental data with theoretical and computational studies.
- Data Integrity: Storing structural data in SWC format ensures that the data is not subjected to the loss of information that can occur with image compression or the variability introduced by different image acquisition techniques.

1.2. Details on the pre-training strategies of MorphRep

The model contains teacher network and student network. Each network is based on graph transformers as described in Section 2.3. Denote the input neuron graph as $(\mathbf{V}, \mathbf{A})$ where $\mathbf{V} = \{v_i, \mathbf{x}_i\}_{i=1}^n$ is the input nodes ($\mathbf{x}_i$ is the feature of v_i) and $\mathbf{A}$ is the adjacency matrix of the input neuron graph. The input is first passed to the data augmentation module using the strategies describe in Section 2.2. The teacher encoder network and student encoder network are denoted as F_{teacher} and F_{student} respectively. There are M neurons in total within one batch. The output embeddings of teacher network can be represented as $\mathbf{y}_{\text{teacher}} = F(\mathbf{V}, \mathbf{A})$. The output $[CLS]$ token embeddings of teacher and student network $[CLS]$ token are denoted as $\mathbf{y}_{\text{teacher}}[CLS]$ and $\mathbf{y}_{\text{student}}[CLS]$. Similarly, The output ith token embeddings of teacher and student network ith token are denoted as $\mathbf{y}_{\text{teacher}}[i]$ and $\mathbf{y}_{\text{student}}[i]$. The model is trained by multiple loss terms, including consistency loss, reconstruction loss and KoLeo regularization loss (Sablayrolles et al., 2019). The details of each loss are shown as follows.

Consistency Loss

The consistency loss aims to maximize the consistency between the output $[CLS]$ token embeddings from the teacher network and student network to enable better representation learning. Concretely, consistency loss contains global consistency loss and local consistency loss. To compute the global consistency loss, we first perform a global cropping of neurons. Concretely, we ensure the integrity of soma points in $\mathbf{V}$ and downsample the number of neurons. We denote the cropped view of neuron as $\mathbf{V}_c$. Then, the cropped view of neuron is passed to the data augmentation module and obtain two augmented view of data, namely $\{\mathbf{V}_{DA1}, \mathbf{A}_{DA1}\}$ and $\{\mathbf{V}_{DA2}, \mathbf{A}_{DA2}\}$. Then, we obtain the teacher network output as $\mathbf{y}_{\text{teacher}} = F(\mathbf{V}_{DA1}, \mathbf{A}_{DA1})$ and the student network output as $\mathbf{y}_{\text{student}} = F(\mathbf{V}_{DA2}, \mathbf{A}_{DA2})$. The cross-entropy loss between $\mathbf{y}_{\text{teacher}}[CLS]$ and $\mathbf{y}_{\text{student}}[CLS]$ is used as the global consistency loss. For the local consistency loss, first, all axons in the input neuron graph are removed. Then, one random connected component in the graph $\mathbf{V}'$ is selected and used as input to the student network, denoted as $\mathbf{y}_{\text{student-local}} = F_{\text{student}}(\mathbf{V}', \mathbf{A}')$. The cross-entropy loss between student and teacher network output $[CLS]$ embeddings, $\mathbf{y}_{\text{student-local}}[CLS]$ and $\mathbf{y}_{\text{teacher}}[CLS]$, is used as local consistency loss. We apply Sinkhorn-Knopp centering (Caron et al., 2020) to the teacher network output following (Oquab et al., 2023). The global consistency and local consistency losses are combined as the final consistency loss.

Reconstruction Loss

We randomly replace the nodes in the input graph with [MASK] token or random node features with certain probability, with the same strategy adapted in (Hou et al., 2022). The masked node index list is denoted as B. Concretely, the masked input graph can be represented as $(\mathbf{V}_{\text{mask}}, \mathbf{A})$ where the features of some nodes $\{v_i\}_{i \in B}$ in $\mathbf{V}_{\text{mask}}$ are replaced by [MASK] token embedding $\mathbf{x}_{[\text{MASK}]}$. The [MASK] token embeddings $\mathbf{x}_{[\text{MASK}]}$ are also trainable parameters in the network. The adjacency matrix $\mathbf{A}$ is not affected by the masking process. Then, we feed the masked input $(\mathbf{V}_{\text{mask}}, \mathbf{A})$ to the student network and the original input $(\mathbf{V}, \mathbf{A})$ to the teacher network, denoted as $\mathbf{y}_{\text{teacher}} = F_{\text{teacher}}(\mathbf{V}, \mathbf{A})$ and $\mathbf{y}_{\text{student}} = F_{\text{student}}(\mathbf{V}_{\text{mask}}, \mathbf{A})$. The output embeddings of the [MASK] tokens ($\mathbf{y}_{\text{student}}[B]$) are used to compute cross-entropy loss with corresponding positions in the teacher network output, whose input graph is not masked ($\mathbf{y}_{\text{teacher}}[B]$). Adding reconstruction loss enables better representation learning at the node level and subgraph level, which complements with the consistency loss to learn robust graph embeddings at multiple levels.

KoLeo Regularization Loss

The Koleo regularization loss is designed to encourage a uniform span of the features within a batch. To be more specific, given a batch of M output $[CLS]$ token embeddings from the student encoder network $\{F_{\text{student}}(\mathbf{V}_i, \mathbf{A}_i)[CLS]\}_{i=1}^{M}$, KoLeo loss is defined as $L_{koleo} = -\frac{1}{n}\sum_{i=1}^{n} log(d_{n,i})$, where $d_{n,i} = \min_{j \neq i} ||F_{\text{student}}(\mathbf{V}_i, \mathbf{A}_i)[CLS] - F_{\text{student}}(\mathbf{V}_j, \mathbf{A}_j)[CLS]||$ is the minimal distance between $F_{\text{student}}(\mathbf{V}_i, \mathbf{A}_i)[CLS]\}$ and any

other student network [CLS] embedding within the batch. $F_{\text{student}}(\mathbf{V}_i, \mathbf{A}_i)[CLS]$ is also normalized before computing Koleo Regularization loss.

1.3. Details of the hyperparameters in pre-training

For pre-training, we set the number of layers to 7, the number of attention heads to 11 and the embedding size to 128. We keep the ratio between consistency loss, reconstruction loss and KoLeo regularization loss to 10:10:1. To pre-train MorphRep, we utilized 8 Nvidia GeForce RTX 3090 GPUs for 30 epochs, which took approximately 30 hours. The base learning rate was set to 0.003, and the minimum learning rate was set to 0.000001. We employed the AdamW optimizer with learning rate scheduling. The embedding size was set to 128, and the hidden dimension of the attention head was set to 512. During the masking process of the reconstruction loss, we sampled the mask ratio between 0.1 and 0.5. We replaced the [MASK] token with random nodes in the neuron morphology graph with a probability of 0.1. The batch size used per GPU was set to 8. Further details regarding model training configurations have been made available alongside the codebase.

1.4. Details on the fine-tuning strategies

The adapter contains two linear layers (one for down projection and the other for up projection) and non-linear activation function ReLU. The input (embedding shape 768) is first passed to the down projection layer and projected to low-dimensional embedding (embedding shape 64), then passed to non-linear activation function and projected back to the original dimension through the up-projection layer.

1.5. Details on the neuron morphometrics

Depth: Depth refers to the distance between the neuron's soma (cell body) and the furthest point along its dendritic arbor. It provides information about the spatial extent of the neuron's dendrites. **Fragmentation**: Fragmentation refers to the degree to which the dendritic arbor of a neuron is divided or fragmented. It indicates the presence of discontinuities or breaks in the dendritic tree. **Length**: Length refers to the total linear extent of the dendrites of a neuron. It is measured as the sum of all dendritic segments. **Volume**: Volume represents the three-dimensional space occupied by the neuron's dendritic arbor. It provides an estimate of the overall size or mass of the dendritic tree. **Bifurcation Amplification Local (Bif ampl local)**: Bifurcation amplification local describes the local complexity of the dendritic branching pattern. It quantifies the degree of branching and the number of dendritic segments emerging from a particular point. **Diameter**: Diameter refers to the width or thickness of the dendrites. It provides information about the size of the dendritic branches. **Number of Stems**: The number of stems refers to the count of major dendritic branches emerging directly from the soma. Each stem represents a primary branch that gives rise to further dendritic branches. **Path Distances**: Path distances represent the lengths of specific paths or segments within the dendritic tree. They indicate the distance between two points along the dendritic arbor. **Width**: Width refers to the lateral extent of the dendrites. It represents the maximum distance between the leftmost and rightmost points of the dendritic tree. **Surface**: Surface refers to the total area of the neuron's dendritic arbor. It quantifies the amount of membrane surface available for synaptic connections. **Soma Surface**: Soma surface denotes the surface area of the neuron's cell body. It provides information about the area available for receiving inputs from other neurons. **Euclidean Distance**: Euclidean distance measures the straight-line distance between two points in the three-dimensional space of the dendritic arbor. It provides a direct measure of spatial separation. **Branch Order**: Branch order represents the hierarchical level or generation of a dendritic branch within the dendritic tree. Branch order increases with each level of branching away from the soma.

1.6. Detailed information related to CAJAL and MorphVAE

MorphVAE: For each neuron in each dataset, a random walk of length 32 (3-D) is performed for 32 iterations. As a result, the shape of the features obtained from the random walk in MorphVAE is $(N, 32, 32, 3)$, which is then reshaped to $(N \times 32, 32, 3)$. The label of each random walk sample remains the same as the original sample. The VAE training settings include a latent embedding (neuron morphology representation) of size 32, a dropout rate of 0.1, a pooling method of 'max', and a learning rate of 0.01. The training is conducted for 500 iterations, with the loss being the Mean Squared Error (MSE) between the reconstructed random walk and the original walk.

CAJAL: We uniformly sample 100 points on each neuron and calculate the GW distance distribution. Based on the distance distribution, we compute the distance for each neuron, resulting in a matrix of shape (N, N) that represents the samples' representation. Therefore, it can be observed that CAJAL may face challenges when scaling to larger datasets, as the dimension of the neuron representation depends on the dataset size.

1.7. Discussion on the effect of Hyperparameters in UMAP

We varied the parameters of UMAP for Fig.4 and analyze the impact of UMAP parameters on visualization. We varied the n-neighbors and min-dist parameters of the UMAP algorithm and visualized the neuron morphology embeddings. The min-dist changes from 0.1,0.2,0.3,0.4 to 0.5 and the n-neighbors changes from 5,10,20,30 to 40. The results are shown respectively in Supplementary Fig. 5-9. We can see that the results and observations are generally consistent across different hyper-parameter settings, which demonstrates the generalizability and stability of our method.

1.8. Ablation Study

We conducted an analysis to evaluate the effectiveness of the modules used in MorphRep pre-training and attribute the contributions of each component. Due to the time-consuming and resource-intensive nature of pre-training, we focused on assessing the main modules rather than conducting a comprehensive evaluation of each individual component. Initially, we trained the base model exclusively with the global consistency loss, resulting in sub-optimal performance. However, when we introduced the reconstruction loss and the local consistency loss as additional components, we observed a significant improvement in the model's performance. This finding emphasizes the necessity of incorporating local information in MorphRep training. Additionally, we experimented with a simple yet effective technique of adding random noise to the [MASK] token. Specifically, we replaced the [MASK] token with a random node in

Table 1. Ablation Study of MorphRep

Setting\Dataset	BBP	BIL		M1-EXC		ACT		Average
Label	Cell Type	Cell Type	Brain Region	Cell Type	Brain Region	Cell Type	Brain Region	
Consistency (Global)	0.463	0.555	0.686	**0.710**	0.706	0.677	0.484	0.612
+ Reconstruction Loss	0.805	0.738	0.787	0.692	0.703	0.927	0.628	0.754
+ Consistency (Local)	0.813	0.769	0.794	0.679	0.721	**0.955**	0.660	0.770
+ Noisy Mask	**0.822**	**0.804**	**0.802**	0.706	**0.736**	0.940	**0.662**	**0.782**

the neuron. This modification resulted in substantial performance improvement.

2. Supplementary Figures

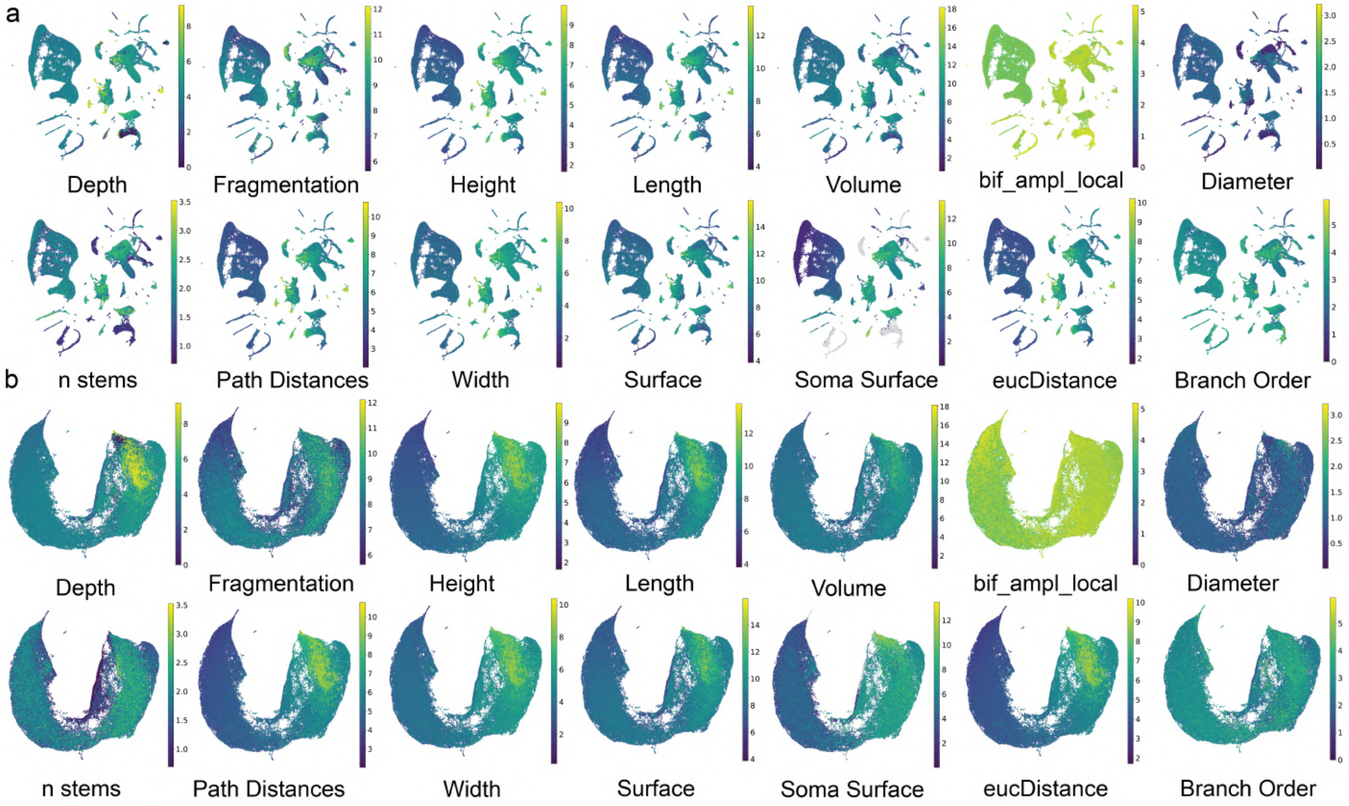

Supplementary Figure 1: The characterization of MorphRep and GraphDino-P on neuron shape space. UMAP visualization of pre-trained MorphRep and GraphDino-P embeddings with respect to a wide range of neuron morphometric features. a. MorphRep. b. GraphDino-P

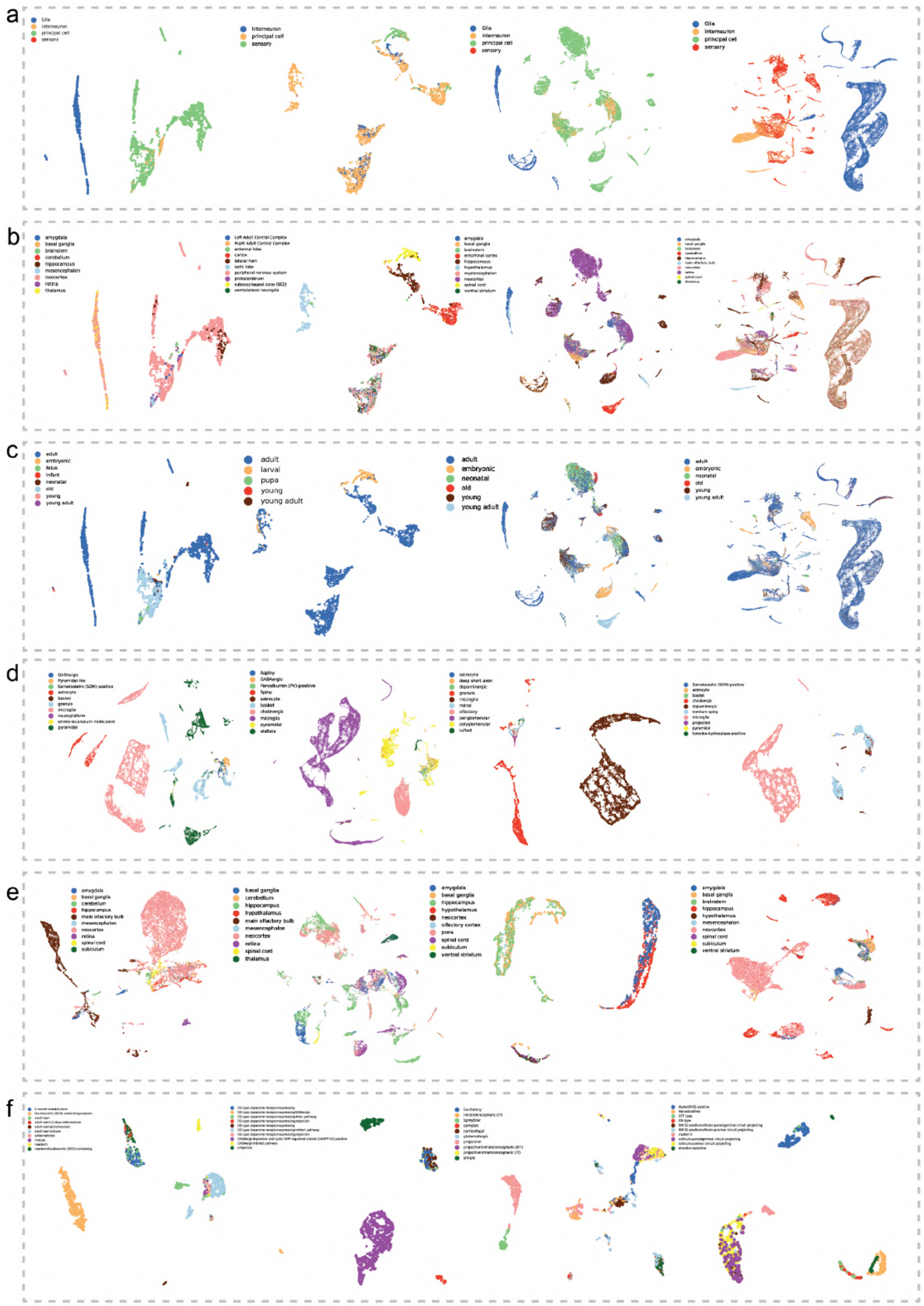

Supplementary Figure 2: UMAP visualization of neuron morphology embeddings generated by MorphRep. (A)(B) denotes the visualization of neurons in A colored by B. **a**.(Human, Drosophila,rat, mouse)(primary cell type) **b**.(Human, Drosophila,rat, mouse)(primary region) **c**.(Human, Drosophila,rat, mouse)(age) **d**.(mouse hippocampus, mouse neocortex, mouse main olfactory bulb, mouse basal ganglia)(secondary cell type) **e**.(mouse interneuron, mouse principal cell, rat Glia, rat principal cell)(primary brain region) **f**.(mouse principal cell-granule cell, mouse principal cell-medium spiny cell, mouse principal cell-pyramidal cell, mouse principal cell-ganglion cell)(tertiary cell type).

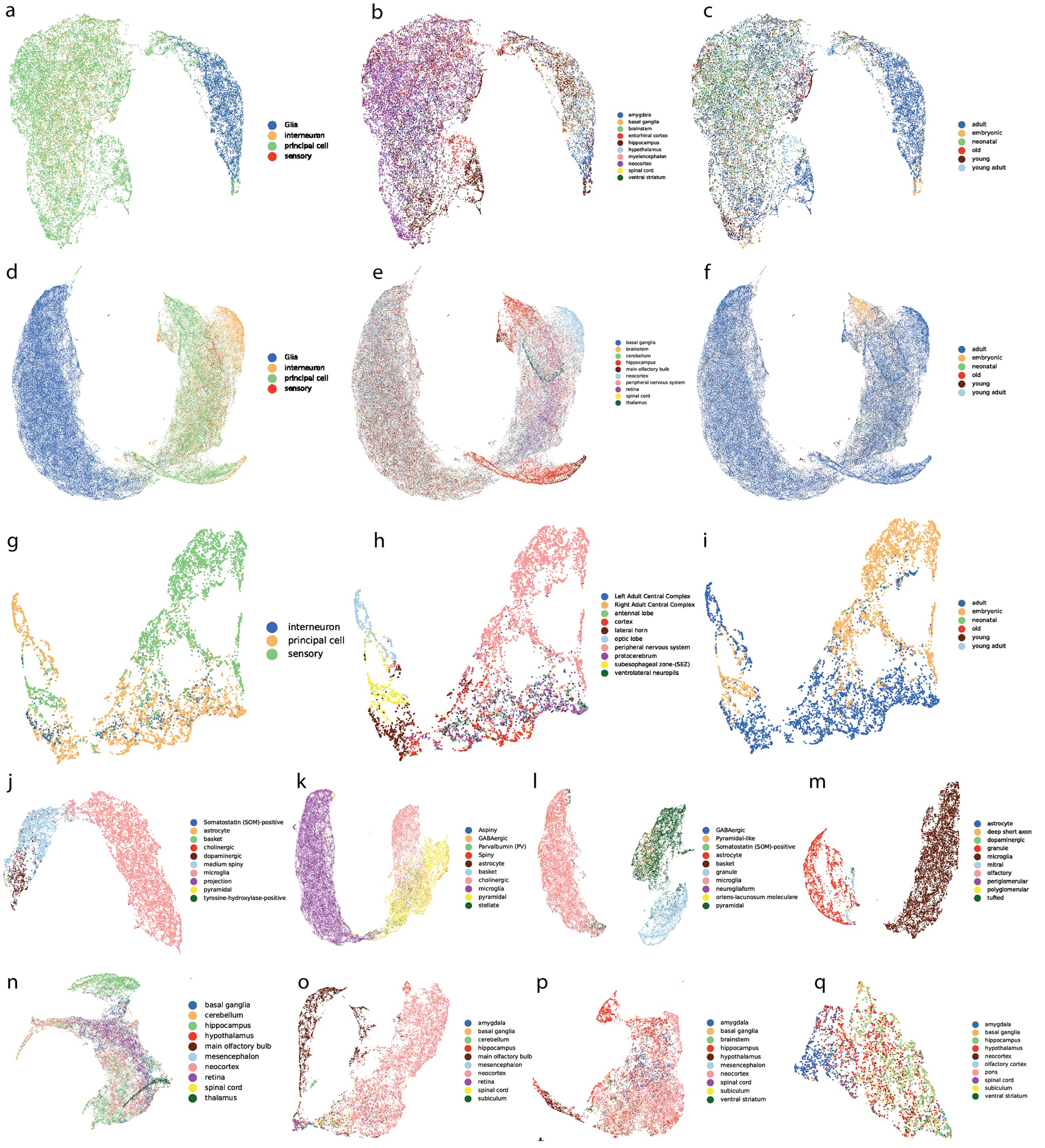

Supplementary Figure 3: UMAP visualization of neuron embeddings generated by GraphDino-P. A(B) denotes the visualization of neurons in A colored by B. **a-c** Rat(primary cell type, primary brain region, age) **d-f** Mouse(primary cell type, primary brain region, age) **g-i** Drosophila(primary cell type, primary brain region, age) **j-m** (mouse basal ganglia, mouse neocortex, mouse hippocampus, mouse main olfactory bulb) secondary cell type **n-q** (mouse principal cell, mouse interneuron, rat principal cell, rat Glia) primary brain region

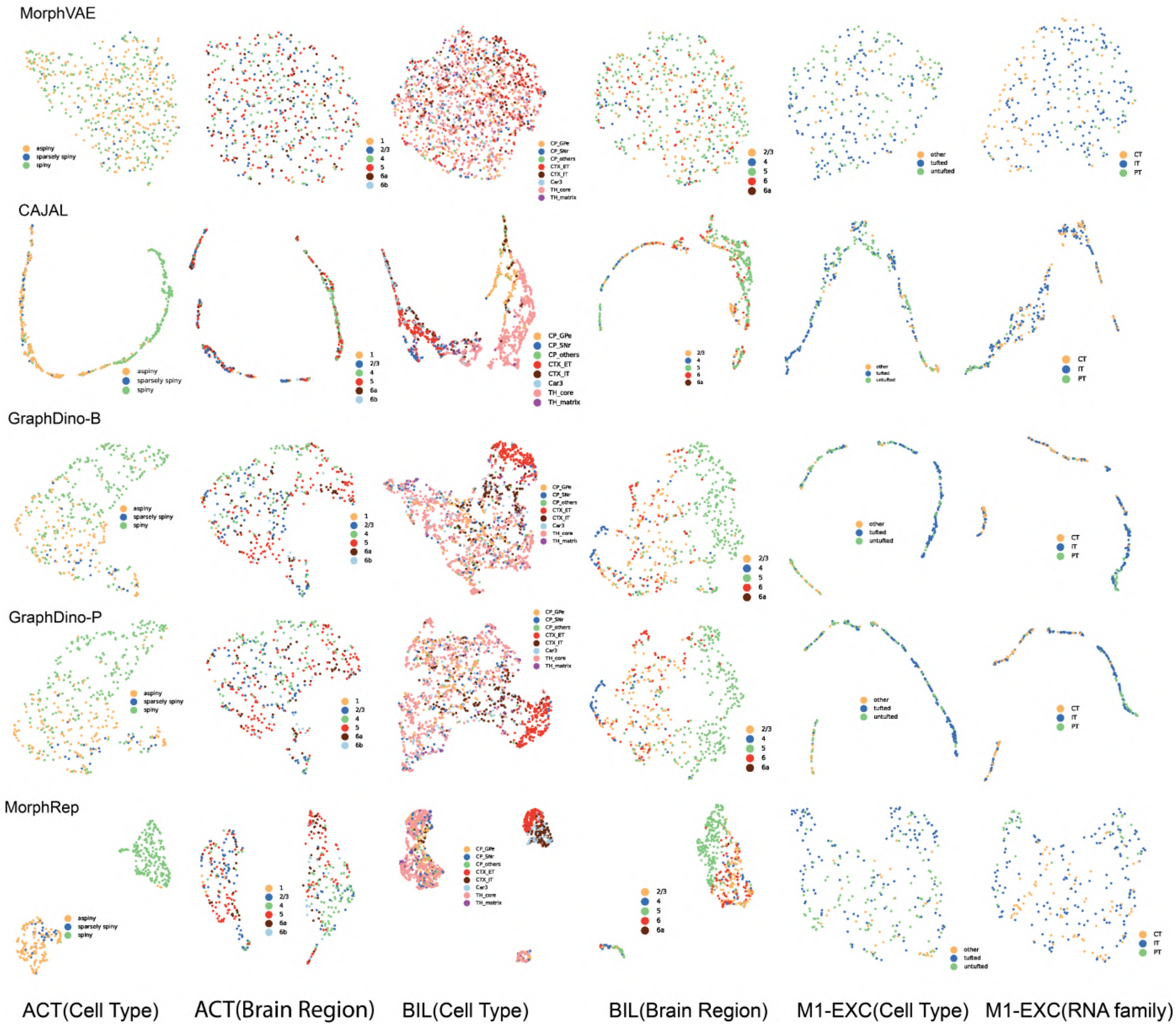

Supplementary Figure 4: UMAP visualization of neuron embeddings generated on six benchmarking datasets with MorphVAE, CAJAL, GraphDino-B, GraphDino-P and MorphRep.

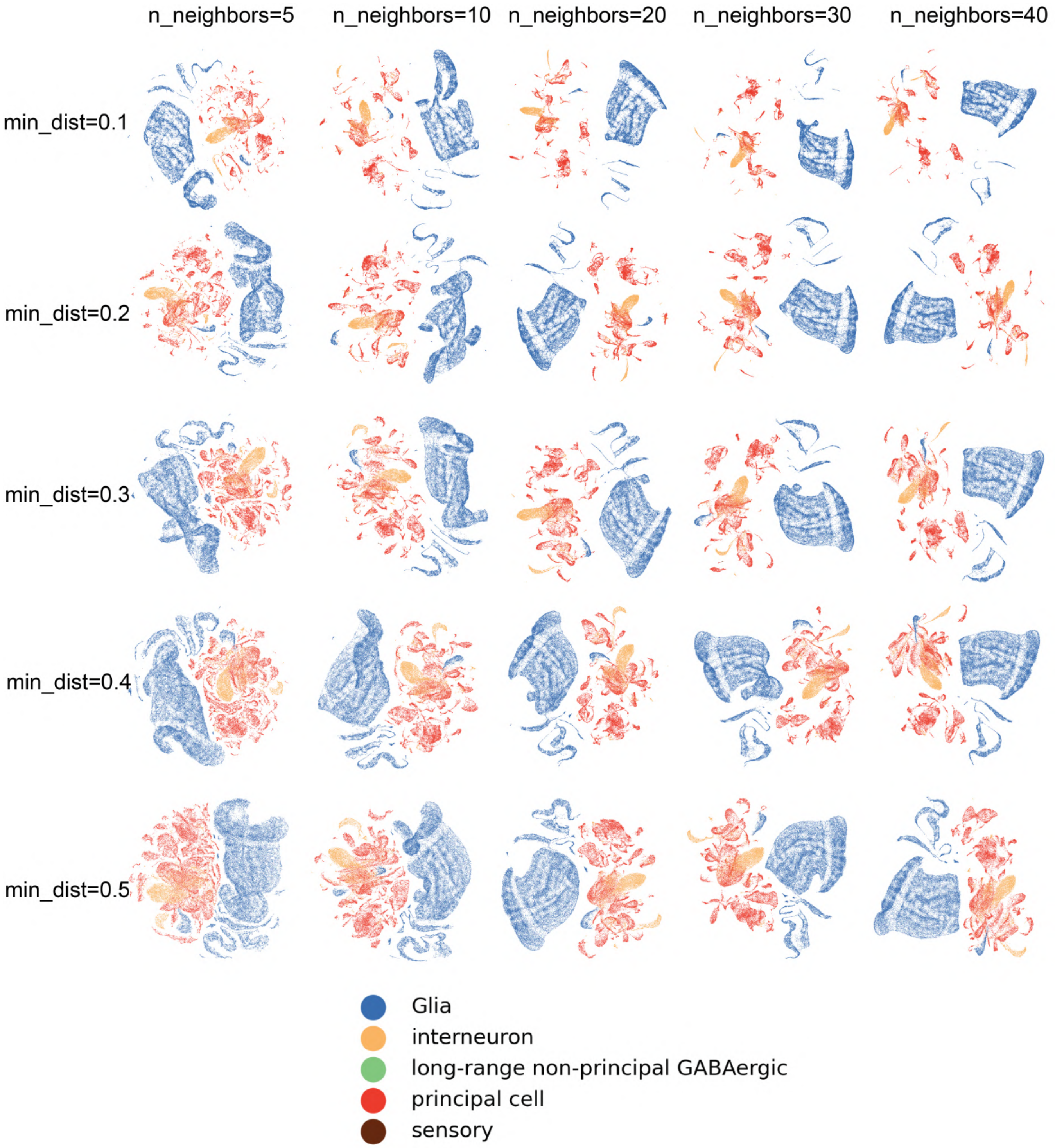

Supplementary Figure 5: Analysis of the hyperparameter effect on the UMAP visualization (Mouse, Primary Cell Type)

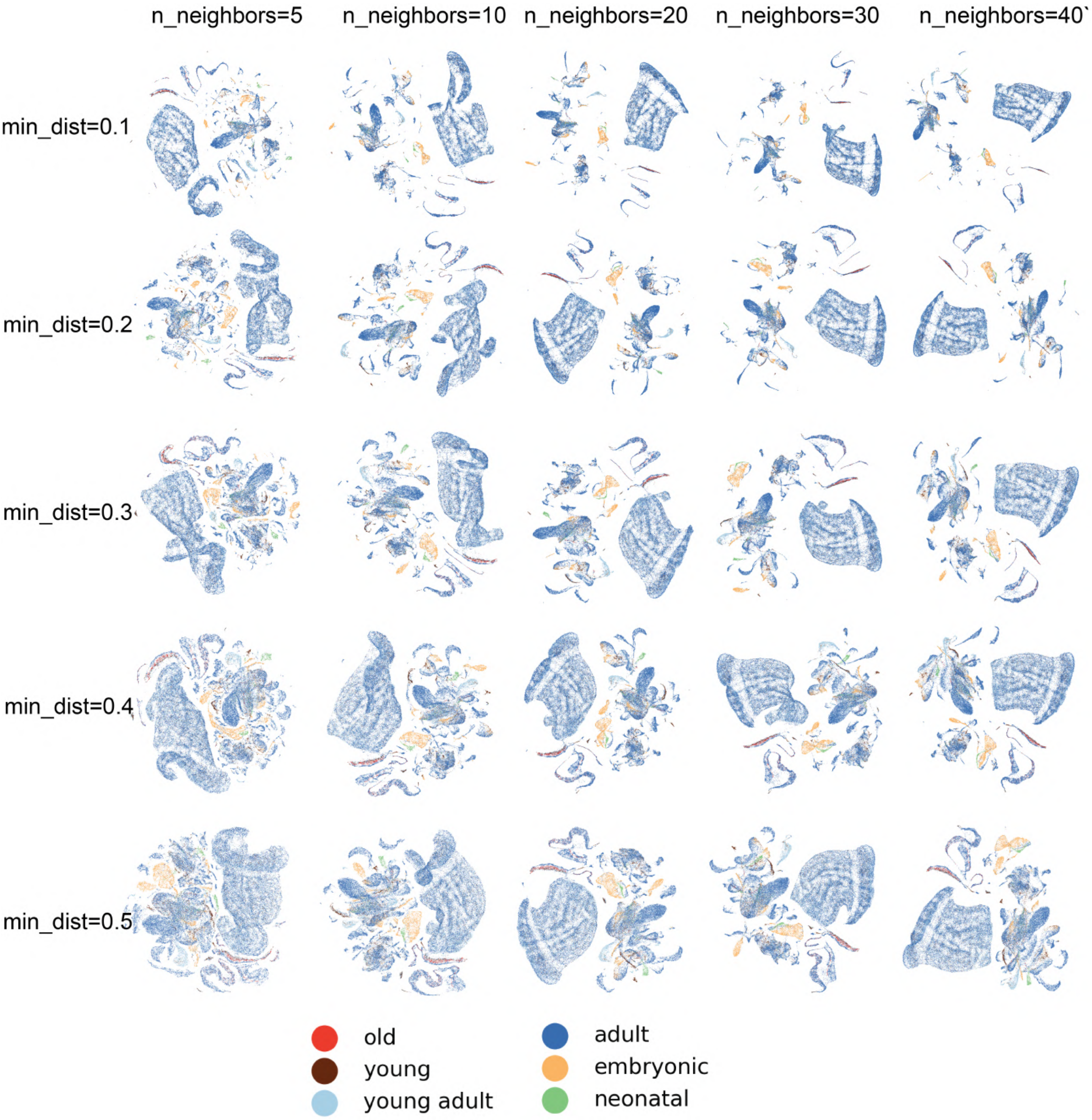

Supplementary Figure 6: Analysis of the hyperparameter effect on the UMAP visualization (Mouse, Age)

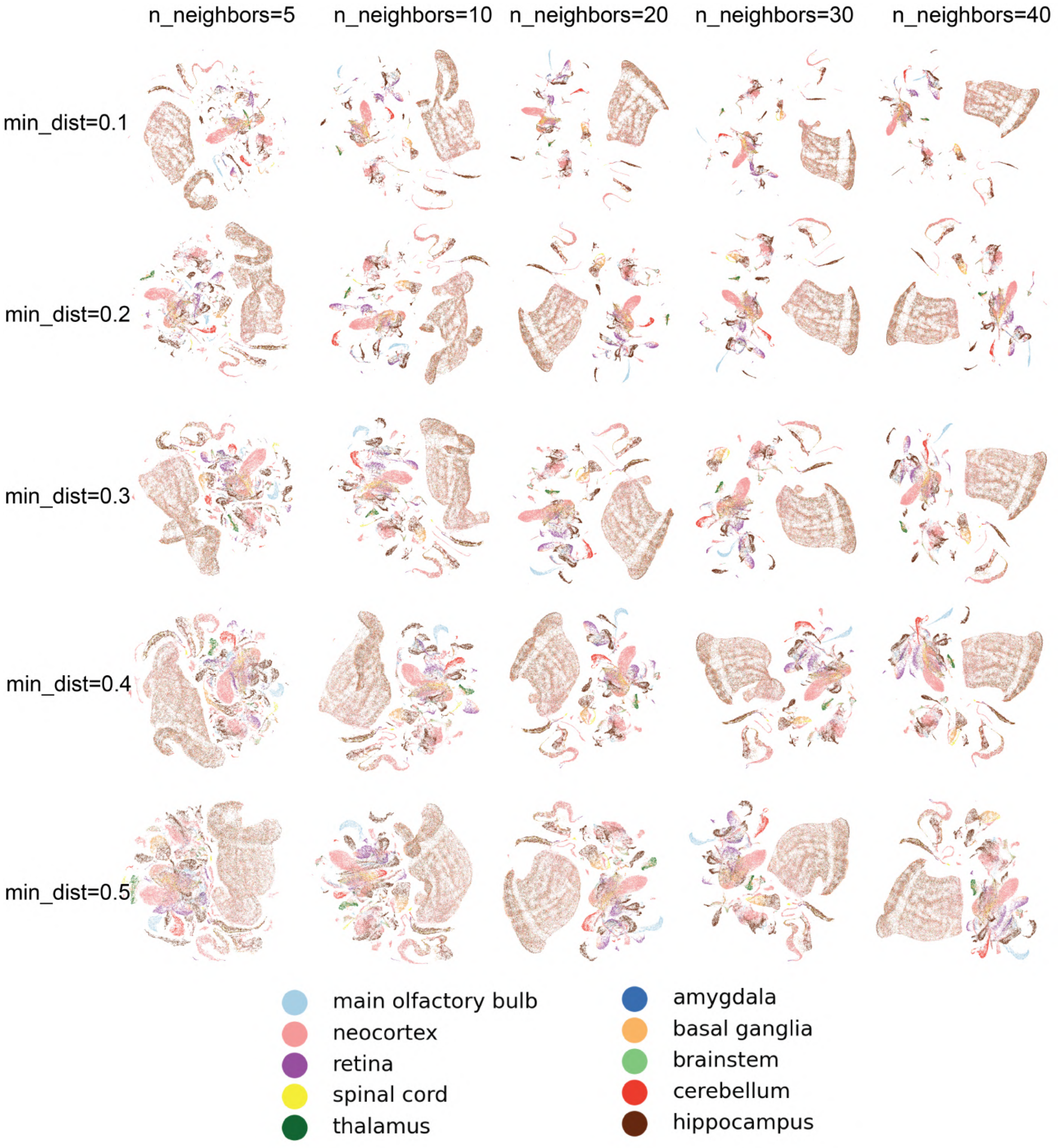

Supplementary Figure 7: Analysis of the hyperparameter effect on the UMAP visualization (Mouse, Brain region)

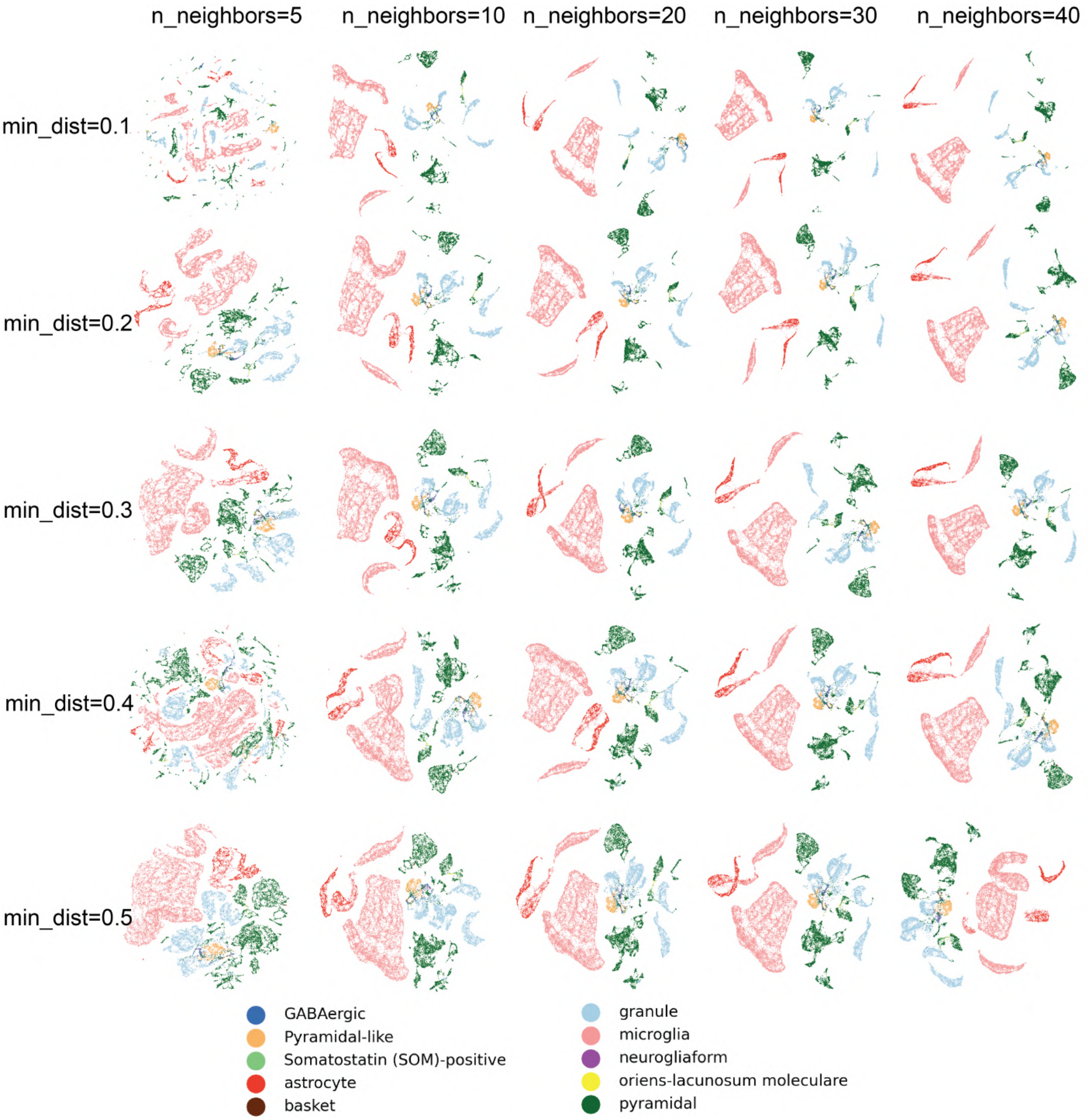

Supplementary Figure 8: Analysis of the hyperparameter effect on the UMAP visualization (Mouse Hippocampus, Secondary cell type)

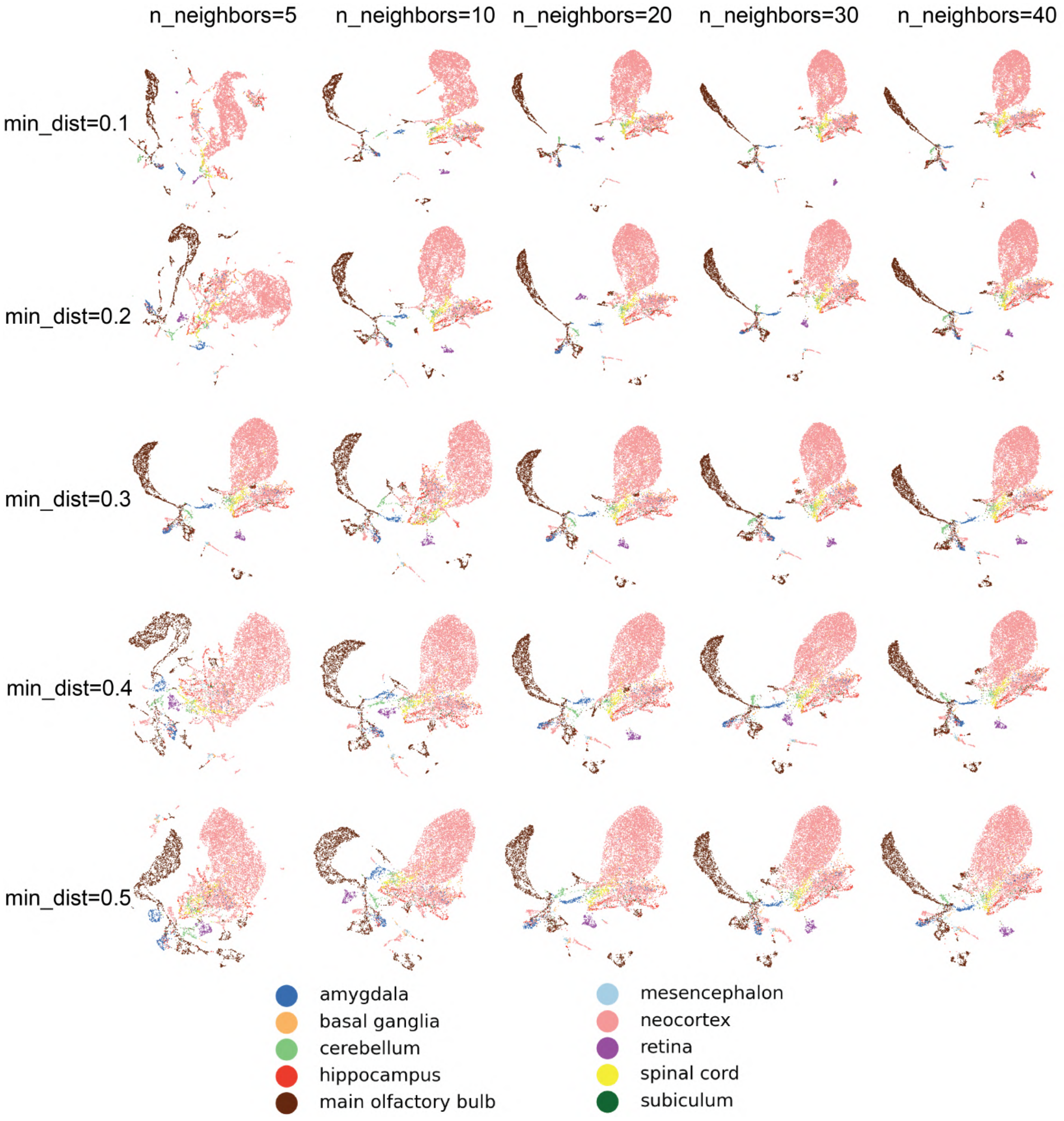

Supplementary Figure 9: Analysis of the hyperparameter effect on the UMAP visualization (Mouse Interneuron, Primary brain region)

3. Supplementary Tables

Table 2. Label distribution of downstream datasets in this paper

Dataset	Label Distribution							
ACT(Cell Type)	spiny	aspiny	sparsely spiny					
	234	231	40					
ACT(Brain Region)	5	4	2 or 3	6a	6b	1		
	164	123	107	75	21	15		
BIL(Cell Type)	Car3	CTX_IT	CP_GPe	CP_others	CP_SNr	TH_matrix	TH_core	CTX_ET
	102	176	180	31	100	62	637	210
BIL(Brain Region)	6	5	4	2/3	6a			
	83	260	53	117	1			
M1-EXC(Cell Type)	untufted	tufted	other					
	107	135	33					
M1-EXC(Brain Region)	CT	IT	PT					
	78	155	34					
BBP	55 label annotations in total							

Table 3. Description on neuron morphology disease dataset

Condition	Num.(ratio)
Disease	
**Dichlobenil + Minocycline**	
Control	278(23.88%)
Dichlobenil + Minocycline	618(53.09%)
lipopolysaccharide injection	268(23.02%)
**epilepticus**	
Control	31(20.81%)
Status epilepticus	118(79.19%)
**artery-occlusion**	
Control	88(53.99%)
Middle cerebral artery occlusion	75(46.01%)
**immune-activation**	
Control:	141(75.0%)
Maternal immune activation:	47(25.0%)
**lipopolysaccharide**	
Control	90(47.62%)
lipopolysaccharide injection	99(52.38%)

Table 4. Description on neuron morphology drug dataset

Condition	Num.(ratio)
Drug Injection	
**Salmeterol**	
Control	222(49.89%)
Salmeterol	223(50.11%)
**Cocaine**	
Cocaine	79(52.67%)
Control	71(47.33%)
**Corticosterone**	
Control	181(54.19%)
Corticosterone	153(45.81%)
**DFO-treatment**	
Chronic deferoxamine (DFO)-treatment	291(72.39%)
Control:111	111(27.61%)
**EPEC-treatment**	
Control	56(33.73%)
Enteropathogenic Escherichia coli (EPEC) treated	110(66.27%)

Table 5. Description on neuron morphology environment condition dataset

Condition	Num.(ratio)
Environment	
**Wheel running**	
Control:	129(53.31%)
Wheel running	113(46.69%)
**Microbead occlusion**	
Control	110(54.73%)
Microbead occlusion	91(45.27%)
**Unpredictable Stress**	
Chronic Unpredictable Stress	40(13.11%)
Chronic Unpredictable Stress + unstressed recovery	205(67.21%)
Control	**60(19.67%)**
**Environmental Enrichment**	
Control	160(50.16%)
Environmental enrichment	159(49.84%)
**Chronic Stress**	
Chronic Stress	172(46.11%)
Control	201(53.89%)

Table 6. Description on neuron morphology genetic perturbation condition dataset

Condition	Num.(ratio)
Genetic Perturbation	
**D2R-overexpression**	
Control	102(64.97%)
D2R overexpression	55(35.03%)
**Bmp Overexpression**	
Bmp15 overexpression	30(1.57%)
Bmp2 overexpression	27(1.42%)
Bmp4 overexpression	43(2.25%)
Bmp4 overexpression + Limk1 knockout	20(1.05%)
Bmp5 overexpression	95(4.98%)
Bmp6 overexpression	24(1.26%)
Bmp7 overexpression	27(1.42%)
Bmp8a overexpression	20(1.05%)
Bmpr2 knockout	388(20.35%)
Bmpr2 knockout + Bmpr2 rescue	16(0.84%)
Bmpr2 knockout + Bmpr2d Tail rescue	63(3.3%)
Bmpr2 knockout + Bmpr2dEC rescue	21(1.1%)
Bmpr2 knockout +Bmpr2d Kinase rescue	10(0.52%)
Cdc42 overexpression	9(0.47%)
Control	361(18.93%)
Gdf5 overexpression	36(1.89%)
Gdf6 overexpression	19(1.0%)
Gdf7 overexpression	16(0.84%)
Gdf9 overexpression	10(0.52%)
Limk1 overexpression	60(3.15%)
Limk1 overexpression + Bmpr2 overexpression	47(2.46%)
Limk1 overexpression + Bmpr2d Kinase overexpression	13(0.68%)
Limk1 overexpression + Bmpr2d lacking 751-813aa	29(1.52%)
Limk1 overexpression + Bmpr2d tail overexpression	53(2.78%)
Limk1 overexpression + Bmpr2dEC overexpression	24(1.26%)
Limk1 overexpression + Slingshot1 overexpression	28(1.47%)
Limk1 overexpression + rtTA + TRE-cofilin1(S3A) expression	24(1.26%)
Noggin overexpression	32(1.68%)
Noggin overexpression + Bax knockout	27(1.42%)
Pak1 overexpression	60(3.15%)
Rac1 Overexpression	86(4.51%)
Rac1 overexpression + Bmpr2 overexpression	16(0.84%)
Rac1 overexpression + Limk1 Knockout	48(2.52%)
Rhoa overexpression	52(2.73%)
Slingshot triple knockout	20(1.05%)
Slingshot1 overexpression	18(0.94%)
rtTA+TRE-mTurquoise2 expression	35(1.84%)
**Pcdhg knockout**	
Control	114(48.93%)
gamma protocadherins (Pcdhg) conditional knockout	119(51.07%)
**Mfsd2a deletion**	
Control	156(46.71%)
conditional deletion of Mfsd2a	88(26.35%)
gene-targeted deletion of Mfsd2a	90(26.95%)
**PTEN knockout**	
Control	46(45.54%)
PTEN Knockout	55(54.46%)

References

C. Bardy, M. Van Den Hurk, B. Kakaradov, J. Erwin, B. Jaeger, R. V. Hernandez, T. Eames, A. Paucar, M. Gorris, C. Marchand, et al. Predicting the functional states of human ipsc-derived neurons with single-cell rna-seq and electrophysiology. *Molecular psychiatry*, 21(11):1573–1588, 2016.

M. Caron, I. Misra, J. Mairal, P. Goyal, P. Bojanowski, and A. Joulin. Unsupervised learning of visual features by contrasting cluster assignments. In H. Larochelle, M. Ranzato, R. Hadsell, M. Balcan, and H. Lin, editors, *Advances in Neural Information Processing Systems*, volume 33, pages 9912–9924. Curran Associates, Inc., 2020. URL `https://proceedings.neurips.cc/paper_files/paper/2020/file/70feb62b69f16e0238f741fab228fec2-Paper.pdf`.

N. V. Chawla, K. W. Bowyer, L. O. Hall, and W. P. Kegelmeyer. SMOTE: Synthetic minority over-sampling technique. *Journal of Artificial Intelligence Research*, 16:321–357, jun 2002. doi: 10.1613/jair.953. URL `https://doi.org/10.1613%2Fjair.953`.

H. Chen, J. Yang, D. Iascone, L. Liu, L. He, H. Peng, and J. Yao. TreeMoCo: Contrastive Neuron Morphology Representation Learning. *Advances in Neural Information Processing Systems*, 35:25060–25073, Dec. 2022. URL `https://proceedings.neurips.cc/paper_files/paper/2022/hash/9f989633ffbd47a83caddacad0f0261f-Abstract-Conference.html`.

J. Devlin, M.-W. Chang, K. Lee, and K. Toutanova. BERT: Pre-training of deep bidirectional transformers for language understanding. In *Proceedings of the 2019 Conference of the North American Chapter of the Association for Computational Linguistics: Human Language Technologies, Volume 1 (Long and Short Papers)*, pages 4171–4186, Minneapolis, Minnesota, June 2019. Association for Computational Linguistics. doi: 10.18653/v1/N19-1423. URL `https://aclanthology.org/N19-1423`.

V. P. Dwivedi, C. K. Joshi, A. T. Luu, T. Laurent, Y. Bengio, and X. Bresson. Benchmarking Graph Neural Networks, Dec. 2022. URL `http://arxiv.org/abs/2003.00982`. arXiv:2003.00982 [cs, stat].

N. W. Gouwens, S. A. Sorensen, J. Berg, C. Lee, T. Jarsky, J. Ting, S. M. Sunkin, D. Feng, C. A. Anastassiou, E. Barkan, et al. Classification of electrophysiological and morphological neuron types in the mouse visual cortex. *Nature neuroscience*, 22(7):1182–1195, 2019.

K. W. Govek, P. Nicodemus, Y. Lin, J. Crawford, A. B. Saturnino, H. Cui, K. Zoga, M. P. Hart, and P. G. Camara. CAJAL enables analysis and integration of single-cell morphological data using metric geometry. *Nature Communications*, 14 (1):3672, June 2023. ISSN 2041-1723. doi: 10.1038/s41467-023-39424-2. URL `https://www.nature.com/articles/s41467-023-39424-2`. Number: 1 Publisher: Nature Publishing Group.

L. Hansel, T. Lüddecke, M. A. Weis, and A. S. Ecker. Morphocc: An implicit generative model of neuronal morphologies. 2023.

Z. Hou, X. Liu, Y. Cen, Y. Dong, H. Yang, C. Wang, and J. Tang. Graphmae: Self-supervised masked graph autoencoders, 2022.

S. C. Laturnus and P. Berens. MorphVAE: Generating Neural Morphologies from 3D-Walks using a Variational Autoencoder with Spherical Latent Space. In *Proceedings of the 38th International Conference on Machine Learning*, pages 6021–6031. PMLR, July 2021. URL `https://proceedings.mlr.press/v139/laturnus21a.html`. ISSN: 2640-3498.

M. Oquab, T. Darcet, T. Moutakanni, H. Vo, M. Szafraniec, V. Khalidov, P. Fernandez, D. Haziza, F. Massa, A. El-Nouby, M. Assran, N. Ballas, W. Galuba, R. Howes, P.-Y. Huang, S.-W. Li, I. Misra, M. Rabbat, V. Sharma, G. Synnaeve, H. Xu, H. Jegou, J. Mairal, P. Labatut, A. Joulin, and P. Bojanowski. Dinov2: Learning robust visual features without supervision, 2023.

H. Peng, P. Xie, L. Liu, X. Kuang, Y. Wang, L. Qu, H. Gong, S. Jiang, A. Li, Z. Ruan, et al. Morphological diversity of single neurons in molecularly defined cell types. *Nature*, 598(7879): 174–181, 2021.

S. Ramaswamy, J.-D. Courcol, M. Abdellah, S. R. Adaszewski, N. Antille, S. Arsever, G. Atenekeng, A. Bilgili, Y. Brukau, A. Chalimourda, et al. The neocortical microcircuit collaboration portal: a resource for rat somatosensory cortex. *Frontiers in neural circuits*, 9:44, 2015.

Y. Roohani, K. Huang, and J. Leskovec. Predicting transcriptional outcomes of novel multigene perturbations with gears. *Nature Biotechnology*, pages 1–9, 2023.

A. Sablayrolles, M. Douze, C. Schmid, and H. Jégou. Spreading vectors for similarity search, 2019.

F. Scala, D. Kobak, M. Bernabucci, Y. Bernaerts, C. R. Cadwell, J. R. Castro, L. Hartmanis, X. Jiang, S. Laturnus, E. Miranda, et al. Phenotypic variation of transcriptomic cell types in mouse motor cortex. *Nature*, 598(7879):144–150, 2021.

P. J. Schubert, S. Dorkenwald, M. Januszewski, V. Jain, and J. Kornfeld. Learning cellular morphology with neural networks. *Nature Communications*, 10(1):2736, June 2019. ISSN 2041-1723. doi: 10.1038/s41467-019-10836-3. URL `https://www.nature.com/articles/s41467-019-10836-3`. Number: 1 Publisher: Nature Publishing Group.

M. A. Weis, S. Papadopoulos, L. Hansel, T. Lüddecke, B. Celii, P. G. Fahey, E. Y. Wang, J. A. Bae, A. L. Bodor, D. Brittain, et al. An unsupervised map of excitatory neurons' dendritic morphology in the mouse visual cortex. *bioRxiv*, pages 2022–12, 2022.

M. A. Weis, L. Pede, T. Lüddecke, and A. S. Ecker. Self-Supervised Graph Representation Learning for Neuronal Morphologies. *Transactions on Machine Learning Research*, Mar. 2023. ISSN 2835-8856. URL `https://openreview.net/forum?id=ThhMzfrd6r`.

C.-C. Wu, J. F. Reilly, W. G. Young, J. H. Morrison, and F. E. Bloom. High-throughput morphometric analysis of individual neurons. *Cerebral Cortex*, 14(5):543–554, 2004.

F. Xiong, P. Xie, Z. Zhao, Y. Li, S. Zhao, L. Manubens-Gil, L. Liu, and H. Peng. Dsm: Deep sequential model for complete neuronal morphology representation and feature extraction. *Patterns*, 5 (1), 2024.

N. Yang, K. Zeng, H. Lu, Y. Wu, Z. Yuan, S. Jiang, J. Wu, Y. Wang, and J. Yan. A synchronized layer-by-layer growing approach for plausible neuronal morphology generation. *arXiv preprint arXiv:2401.09500*, 2024.

J. Zhao, X. Chen, Z. Xiong, Z.-J. Zha, and F. Wu. Graph representation learning for large-scale neuronal morphological analysis. *IEEE Transactions on Neural Networks and Learning Systems*, 2022.

T. Zhu, G. Yao, D. Hu, C. Xie, H. Gong, and A. Li. MorphoGNN: Morphological Embedding for Single Neuron with

Graph Neural Networks, May 2022. URL https://www.biorxiv.org/content/10.1101/2022.05.09.491257v1. Pages: 2022.05.09.491257 Section: New Results.
